# Phononic metastructures with ultrawide low frequency three-dimensional bandgaps as broadband low frequency filter

**DOI:** 10.1038/s41598-021-86520-8

**Published:** 2021-03-30

**Authors:** Muhammad, C. W. Lim

**Affiliations:** 1 City University of Hong Kong Shenzhen Research Institute, Shenzhen, People’s Republic of China; 2 Department of Architecture and Civil Engineering, City University of Hong Kong, Kowloon, Hong Kong SAR People’s Republic of China

**Keywords:** Mechanical engineering, Mechanical properties

## Abstract

Vibration and noise control are among the classical engineering problems that still draw extensive research interest today. Multiple active and passive control techniques to resolve these problems have been reported, however, the challenges remain substantial. The recent surge of research activities on acoustic metamaterials for vibration and noise control are testimony to the fact that acoustic metamaterial is no longer limited to pure theoretical concepts. For vibration and noise control over an ultrawide frequency region, 3-D metastructures emerge as a novel solution tool to resolve this problem. In that context, the present study reports a novel proposal for 3-D monolithic phononic metastructures with the capability to induce low frequency ultrawide three-dimensional bandgaps with relative bandwidth enhancements of 157.6% and 160.1%. The proposed monolithic metastructure designs consist of elastic frame assembly that is connected with the rigid cylindrical masses. Such structural configuration mimics monoatomic mass-spring chain where an elastic spring is connected with a rigid mass. We develop an analytical model based on monoatomic mass-spring chain to determine the acoustic mode frequency responsible for opening the bandgap. The wave dispersion study reveals the presence of ultrawide bandgaps for both types of metastructures. The modal analysis shows distribution of vibration energy in the bandgap opening (*global resonant mode*) and closing (*local resonant mode*) bounding edges. We further analyze the band structures and discuss the physical concepts that govern such ultrawide bandgap. Vibration attenuation inside the bandgap frequency range is demonstrated by frequency response studies conducted by two different finite element models. Thanks to additive manufacturing technology, 3-D prototypes are prepared and low amplitude vibration test is performed to validate the numerical findings. Experimental results show the presence of an ultrawide vibration attenuation zone that spreads over a broadband frequency spectrum. The bandgaps reported by the proposed metastructures are scale and material independent. The research methodology, modelling and design strategy presented here may pave the way for the development of novel meta-devices to control vibration and noises over a broadband frequency range.

Artificial periodic structures that once begin from electromagnetic media are presently hot research topics for vibration and noise control due to their unprecedented dynamic mechanical properties that are inconceivable with respect to natural materials^1,2^. The key property of interest includes formation of bandgap (BG) that is a frequency region where incident wave propagation is prohibited. Although metamaterial waveguiding^3^, focusing and collimation^4^, negative refraction^5^,topological properties^6–8^ and underwater acoustic applications^9–11^ have been explored, the all directions vibration and noise control with ultrawide three-dimensional complete BG is also intriguing. Multiple approaches including monolithic structures^12,13^ and elastic impedance based multi-materials periodic structures through both active^14^ and passive^15–17^ control techniques have been proposed to enlarge the BGs. Among those approaches the recently emerging 3-D phononic structures with complete three-dimensional BG^18–20^, inertial amplification phenomena^12^, actively controlled piezoelectric shunt array technique^14,21,22^, elastic metamaterials with dissipative medium characteristics^16^ and multi-resonant trampoline metamaterials^15^ with trampoline effect^23^ have caught extensive attentions. Apart from these genetic algorithm and topology optimization approaches have been reported to optimize the physical structure and dynamical characteristics of metamaterials^17,24,25^. For instance Lu et al.^17^ developed gradient based optimization technique to maximize the solid-to-void ratio of 3-D phononic structure in order to achieve ultrawide BGs. Recently, artificial intelligence based machine learning and deep learning data-driven methods have also caught enormous attention of phononic community for metamaterial physical structure and mechanical characteristics optimization^26,27^. For instance, Chan et al.^26^ developed a METASET to explore different two-dimensional and three-dimensional shape and property space to optimize the structure of mechanical metamaterials. Besides, the relative bandwidth $\Delta \omega /\omega_{c}$ or gap to mid-gap ratio is among the quantitative measures to determine the performance and robustness of BGs, i.e. a wider BG attenuates wave energy over an ultrawide frequency range^28^. The relative bandwidth $\Delta \omega /\omega_{c}$ of a BG is expressed by $\Delta \omega /\omega_{c} = 2(\omega_{t} - \omega_{b} )/\omega_{t} + \omega_{b}$^28,29^ where $\omega_{t}$ and $\omega_{b}$ are the BG closing and opening bounding edge frequencies, respectively. The abovementioned works with proposed approaches and findings are fascinating. However, substantial issues in term of BG working frequencies, directions and manufacturing of physical structures still exist. For instance, the locally resonant multi-core structures induce wider BGs however the width of BG largely depends upon the mass of resonator/scatterer and impedance mismatch. In order to achieve a wider BG, a larger-sized resonator with material mismatch is required. The design optimization and manufacturing of such composite structures are another challenge.

Recently, 3-D periodic structures consisting of multi-core materials are also proposed to maximize the impedance mismatch in order to achieve wider BGs^17,30^. In such approaches, multi-material based prototyping and/or adjusting the assembly phase of selective materials pose a significant challenge. For a single core structure, this milestone constitutes a significant advancement in terms of structure optimization to adjust the filling ratio between material regions and voids. Likewise, even though the active control technique induces ultrawide vibration attenuation zone^14^, the working temperature and environment greatly impact the performance. The manufacturing of such smart devices is another problem that needs advanced technology. In this regard, the present work proposes two phononic metastructure prototypes that consist of a single core structures (monolithic designs) and it is capable of inducing extremely wide low frequency three-dimensional BG. The proposed metastructure morphology consists of rigid masses and elastic beams/frame assembly resembling the conventional monoatomic mass-spring chain. Such design configuration plays an important role in the birth of ultrawide BG. The physical mechanism behind the enlarged BG is also explained. We found that the ultrawide BGs are induced by the principle of mode separation or modal masses participation^31^ where the *global* and *local* resonant modes and their localized vibrational energy in the unit cell structure is found responsible for opening and closing of the BG. The proposed metastructures are designed in such a manner that all vibrational energy concentrated in the complete unit cell structure results in the opening of BG. While the local resonant mode with vibrational energy surrounding the elastic frame assembly is found effective in closing the BG. In this study, the prior and later resonant modes are refereed as *global* and *local* eigenmodes respectively. In the preceding sections, such terminologies will be used concurrently to explain the presence of ultrawide BG.

The present study is based on a rigorous numerical modelling with experiment tests on proposed metastructures to envisage the vibration attenuation over broadband frequency spectrum. We demonstrated the vibration mitigation capability from the proposed structures by numerical and experimental means. For the details of theoretical framework, governing equations and some preliminary metastructure designs, one can refer to the supplementary information. In this study, first an analytical model is developed to calculate the acoustic mode frequency responsible for opening the BG^1^ and subsequently a finite element based wave dispersion and frequency response studies are performed to investigate the BG and to present the vibration attenuation inside the BG frequency region. By additive manufacturing technology, the 3-D prototypes of proposed metastructures are prepared by using 3-D printer OBJET30 *Strata Sys Ltd* and low amplitude vibration test is performed to corroborate our numerical findings and validate the presence and effectiveness of ultrawide three-dimensional BGs. It is noted that although an arbitrary polymeric material (VeroWhite) is selected for numerical analysis and experiment tests, the ultrawide BG reported in this study is actually scale and material independent. That means, the BG reported by the proposed metastructures is a physical property. The change in material and/or size of unit cell structure will alter the frequency range, however the ultrawide BG property will not change. For the same reason, we present the band structures in general frequency *f* and normalized frequency $f_{nd} = fa/v$ where $v = \sqrt {E/\rho }$ is the longitudinal wave velocity. The 3-D metastructure design approach, research methodology and numerical and experimental findings presented here will likely find potential applications in the vibration and noise control facilities and mechanical systems.

## Prototypes and modal comparison

**Figure 1:**
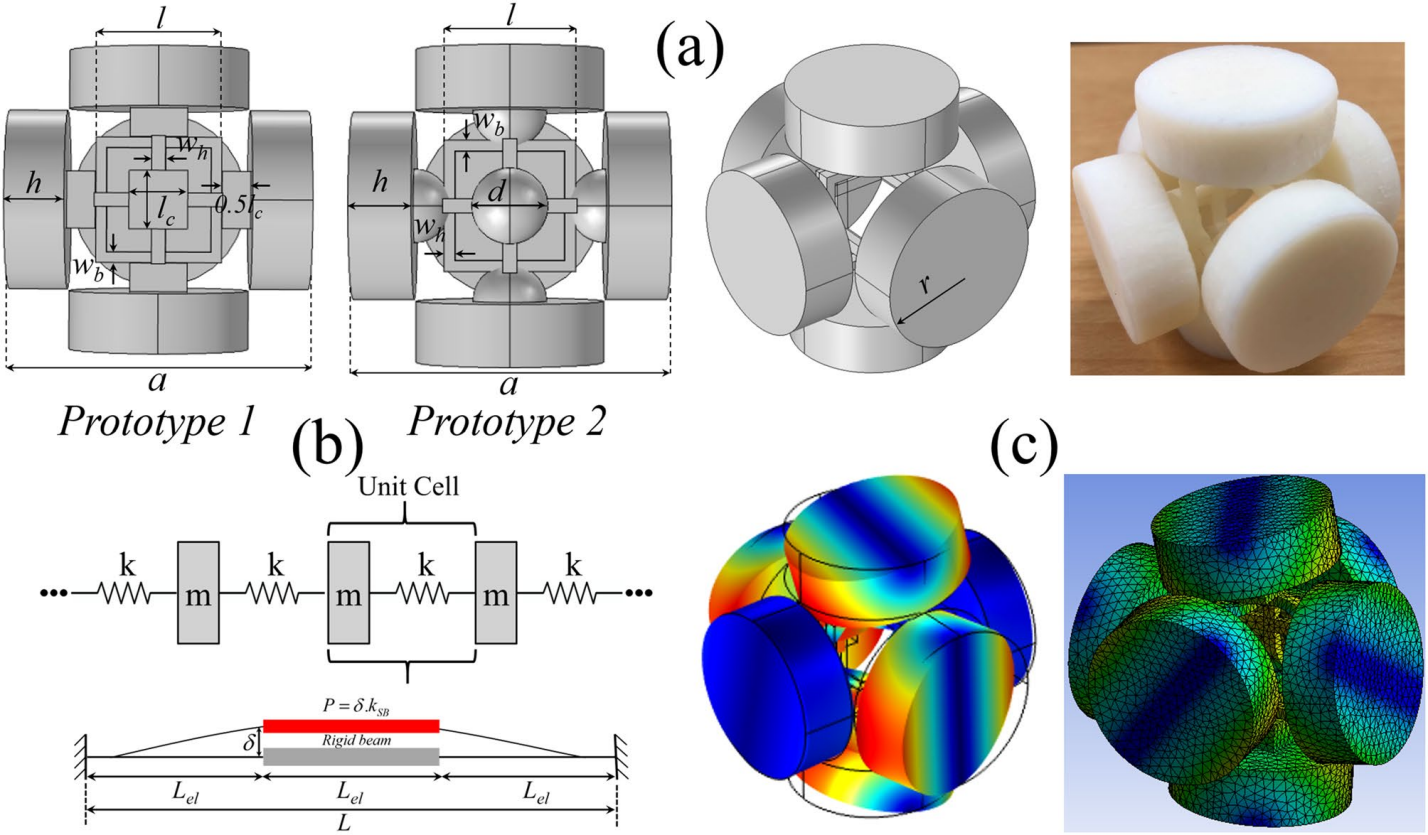
Proposed prototypes for 3-D phononic metastructures. (**a**) Schematic description for prototype 1–2 with 3-D printed sample. (**b**) Monoatomic mass-spring chain along with simplified beam structure. (**c**) Vibration mode for the lower bounding edge of first BG by COMSOL Multiphysics 5.4 (left) and ANSYS 2020 R1 (right). The analytical and FEA results comparison is presented in Table 1.

The opulent topology shown Fig. 1a is schematic diagram of proposed 3-D monolithic phononic metastructures subjected to in-plane elastic wave propagation. In both prototypes, the unit cell topology is realized by an external frame assembly connected with cylindrical masses at the middle points. All the geometric parameters are presented with respect to lattice constant $a = 50\,{\text{mm}}$. The finalized metastructure designs have the following internal properties: frame assembly thickness $w_{b} = 0.035a$, $w_{h} = 0.045a$ that is connected with a cylindrical rigid mass of radius $r = 0.3a$ and height $h = 0.65r$ via a small cube of side length $l_{c} = 0.19a$ for *Prototype 1* or an equivalent volume spherical rigid mass with diameter $d$ for *Prototype 2*. One half of the small cube/sphere is embedded inside the cylindrical mass and the other half is connected to the frame assembly. The small cube/sphere is introduced between the rigid masses and elastic frame assembly for two reasons: (i) it provides strong support between the frame assembly and rigid cylindrical masses; and (ii) it optimizes relative bandwidth $\Delta \omega /\omega_{c}$ that gives a wider BG. For numerical modelling and additive manufacturing, VeroWhite (Young modulus $E = 1.6\,{\text{GPa}}$, mass density $\rho = 1174\,{\text{kg/m}}^{3}$ and Poisson’s ratio $\nu = 0.33$) *Stratasys Ltd* is used. Subsequently dynamic mechanical test is performed to determine the material loss factor $\eta$ that is required for the investigation of effect of material damping on numerically obtained frequency response spectrum/transmission curves, see supplementary information. We obtained the frequency response spectra by FE approach using COMSOL Multiphysics 5.4 and ANSYS 2020 R1. For *Prototype 1* the analytical model and FE results are compared in Table 1. One can observe an excellent agreement between the analytical and numerical models.

**Table 1:** Comparison of different FE models.

	Analytical model	COMSOL multiphysics	ANSYS 2020 R1
Prototype 1	1279.2 Hz (0.05478)	1247.2 Hz (0.05341)	1240 Hz (0.05312)

The modal analysis by both FE codes shows a dominant mixed compressional-bending resonant mode that initiates the BG where the cylindrical masses and small cubes/spheres work as rigid masses while box-like frame assembly exploits the flexural stiffness of the structure when subjected to incident elastic waves. For better understanding, a monoatomic mass-spring chain model is introduced as shown in Fig. 1b. Further details are given in the supplementary information. For a general monoatomic mass-spring chain, the acoustic mode frequency $\omega$ responsible for opening the BG is $\omega = 2\omega_{0}$ where $\omega_{0} = \sqrt {k/m}$ is natural frequency of the system^1^. For the present monoatomic mass-spring chain the acoustic mode frequency calculated is $f_{nd} = 2/2\pi \sqrt {k/m} \left( {a/v} \right)$ that is listed in Table 1. The parameter $m$ incorporates the mass of cylinders and cubes for *Prototype 1* or cylinders and spheres for *Prototype 2* that works as rigid body while parameter $k$ represents the longitudinal stiffness of supporting frame structure with $k = \kappa \gamma k_{SB}$^31^. Here $\kappa$ and $\gamma$ are associated with the stiffness of two sets of beams and the summation of two orthogonal beam stiffnesses, respectively, that are connected with the rigid cylindrical masses via small cubes/spheres. For the present symmetric frame assembly $\kappa = 0.5$ and $\gamma = 2$^31^. Furthermore, as shown in Fig. 1b, for a single beam with the effective length $L_{el} = L/3 - w_{b} /2$ the stiffness of single beam is $k_{SB} = 24EI/L_{el}^{3}$ where $I = \frac{1}{12}w_{b} w_{h}^{3}$ is second moment of area and $L_{el}$ is effective length of beam. The analytical and numerical results comparison for *Prototype 1* is presented in Table 1 and there exists an error of about 2.5–3% between numerical and analytical solutions for the opening bounding edge of the first BG. For *Prototype 2* the BG opening bounding edge obtained from COMSOL Multiphysics and ANSYS 2020 R1 are 929.24 Hz (0.03981) and 939.86 Hz (0.04026), respectively. The vibration modes obtained from COMSOL Multiphysics and ANSYS Workbench is shown in Fig. 1c to double check the accuracy. An excellent agreement can be observed in term of deformation mechanism and displacement field distribution. Further details can be found in the supplementary materials.

## Results and discussion

**Figure 2:**
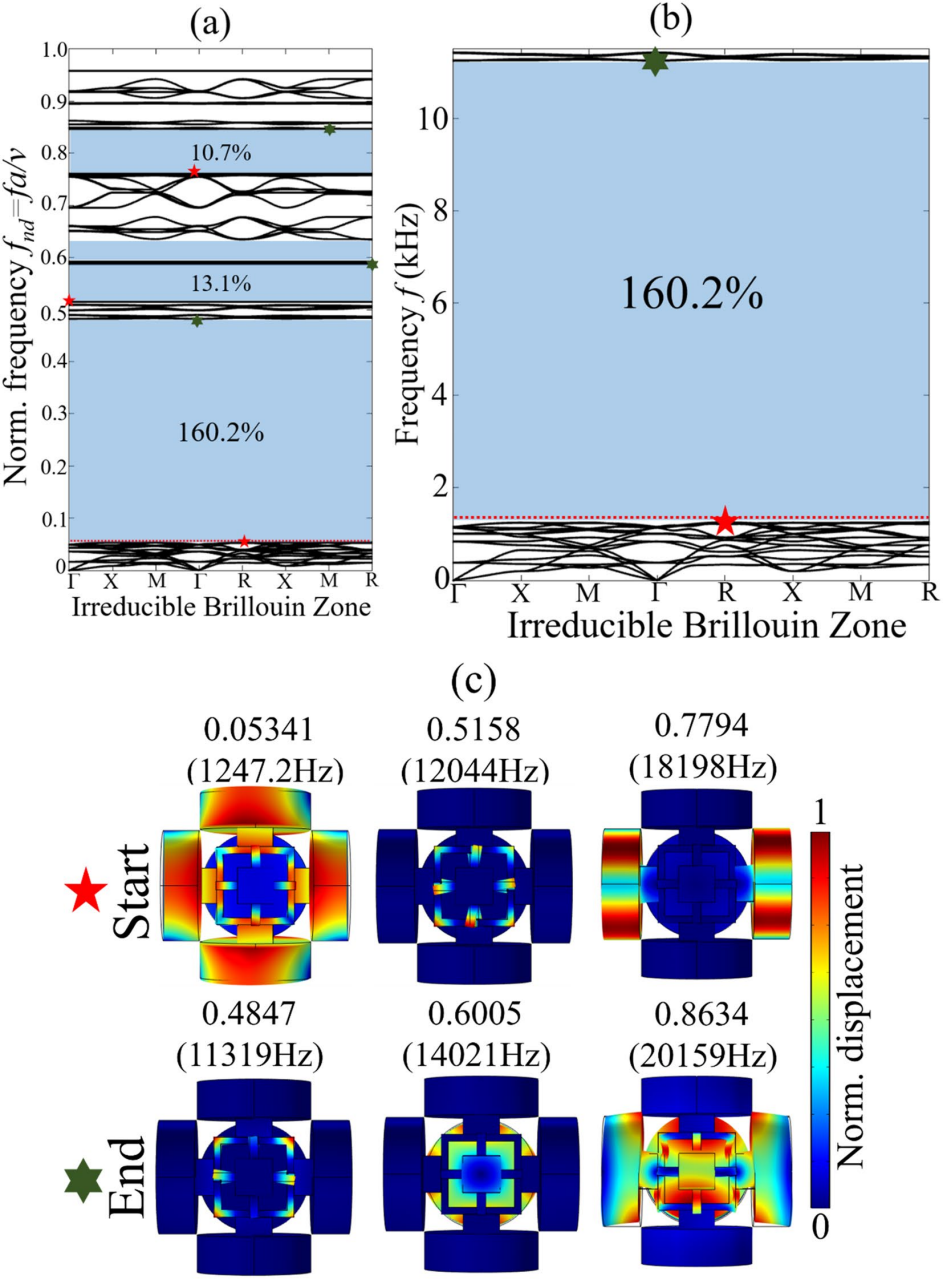
*Prototype 1-* numerical dispersion spectra: (**a**) complete band structure with normalized frequency; (**b**) the widest first BG with $\Delta \omega /\omega_{c}$ of 160.2%; (**c**) vibration modes corresponding to the lower and upper bounding edges of BGs.

For *Prototype 1*, the numerical band structure and BGs determined by COMSOL Multiphysics5.4 is shown in Fig. 2a,b. The boundary of irreducible Brillouin zone is shown at the inset of band structure. The first BG is the widest with the $\Delta \omega /\omega_{c}$ = 160.2%. The vibration modes corresponding to the bounding BG edges are shown in Fig. 2c. The vibration modes associated to BG opening and closing bounding edges are designated with red and green stars, respectively. The monolithic 3-D phononic metastructure proposed here possesses the widest three-dimensional BG with the capability of attenuating mechanical vibration and noises in all three directions.

**Figure 3:**
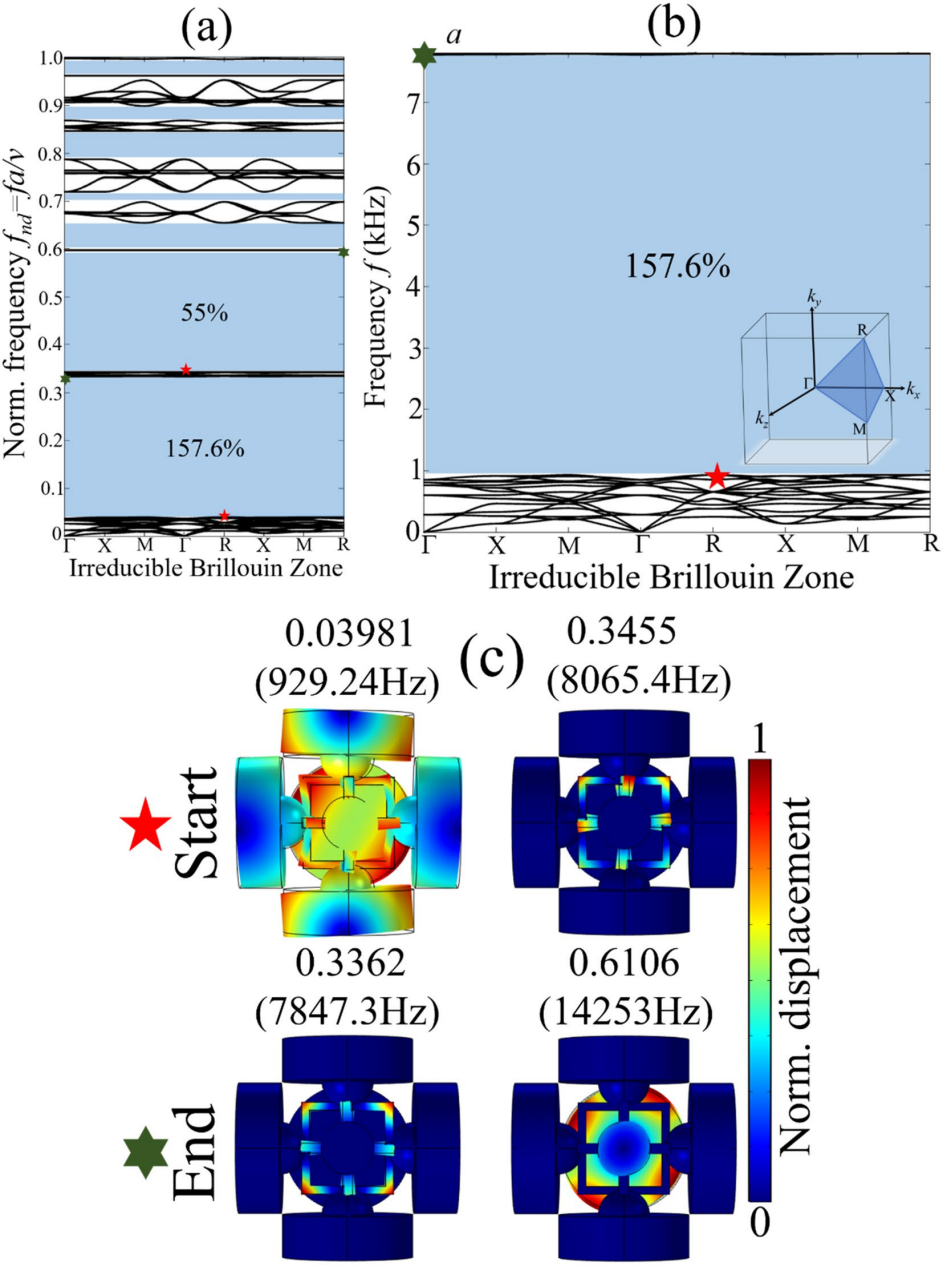
*Prototype 2-* numerical dispersion spectra: (**a**) complete band structure with normalized frequency; (**b**) the widest first BG with $\Delta \omega /\omega_{c}$ of 157.6%; and (**c**) vibration modes corresponding to the lower and upper bounding edges of BGs.

Similarly, the band structure with BGs and vibration modes corresponding to the bounding BG edges for *Prototype 2* are shown in Fig. 3a–c. It is noticed that replacing cube masses with spheres of an equivalent volume resulted in two wide BGs with $\Delta \omega /\omega_{c}$ 157.6% and 55%, respectively. Interestingly both BGs are very close and they are separated by some narrow passbands. The material damping/viscoelasticity effects will weaken this passband that eventually results in a broadband BG covering an extremely wide frequency range^15,28^.

Both *Prototype 1* and *Prototype 2* are design in such a way to maximize the difference between opening and closing bounding edges of the BG. In other words, we tend to optimize the distribution of vibrational energy localized in the different parts of the unit cell structure observed at opening and closing bounding edges of the BGs, see Figs. 2 and 3. Such strategy is refereed as principle of mode separation or modal masses participation. It implies that, for a metastructure unit cell structure abide by periodicity condition, see “Method” the distribution of vibrational energy can be maximized to generate ultrawide BG. In such type of BG, usually two types of resonant modes are involved at the opening and closing bounding edges of the BG. The prior resonant mode is refereed as *global resonant mode* while the later eigenmode is called *local resonant mode*. For example, the metastructure unit cell structures shown in Fig. 1a majorly consist of two components, (i) heavy rigid cylindrical masses with small cubic/spherical masses (ii) thin elastic frame assembly supporting these rigid masses. The band structure shown in Figs. 2 and 3 show that the bounding edges of BGs are characterized by the passbands that is almost uniform throughout the irreducible Brillouin zone. The vibration modes corresponding to these passband enhanced bounding edges demonstrate the confinement of vibrational energy either in the complete unit cell structure (*global resonant mode*) or few parts of the unit cell structure such as elastic frame assembly (*local resonant mode*), see Figs. 2c and 3c. The oscillations of complete unit cell structure including the heavy rigid masses with supporting cubes/spherical masses and elastic frame assembly position the eigenmode to lower frequency regime. This is because, the oscillation of complete unit cell structure enhances the effective mass of resonant system that shift the opening bounding edge of the BG (*global resonant mode*) to lower frequency region. Therefore, in Figs. 2c and 3c the opening bounding edge of BG for *Prototype 1* is positioned at 0.05341(1247.2 Hz) and for *Prototype 2* 0.03981(929.24 Hz). On the other hand, a weaker oscillation of unit cell structure with concentration of vibrational energy inside the elastic frame assembly shift the eigenmode (*local resonant mode*) to far higher frequency i.e. 0.4847 (11,319 Hz) for *Prototype 1* and 0.3362 (7847.3 Hz) for *Prototype 2* compared to the BG opening bounding edge (*global resonant mode*) frequency. This huge difference between *global* and *local* resonant frequencies caused by the metastructure morphology results into generation of ultrawide BG.

In both Figs. 2c and 3c the *global resonant mode* is characterized by a mixed bending and axial motion of complete unit cell structure as shown in Fig. 1c. Since the complete unit cell structure is in robust motion, this placed the eigenfrequency and eigenmode to relatively lower frequency. However, at closing bounding edge of the BG, the rigid masses are at rest and vibrational energy is seemed to be confined in the thin elastic frame assembly only. Since the modal mass contribution by thin elastic frame assembly is much smaller than rigid masses, eventually it shifts the eigenmode to far higher frequency regime. Now any change in geometry of the metastructure either increasing or reducing the size of cylindrical masses and/or cube/spherical masses or thickness of elastic frame assembly will affect the *global* and *local* resonant mode frequencies. A detail analysis is given in the Supplementary information. Thus, this huge difference between *global* and *local* resonant modes in terms of vibrational energy localization and modal masses participation caused by the metastructure morphology at the opening and closing bounding edges of the reported BGs resulted into generation of ultrawide vibration attenuation zones. Any metastructure morphology that support this huge difference of eigenfrequencies with *global* and *local* resonant modes can help induce low frequency ultrawide BG.

In addition, it is found that the *global* and *local* resonant modes are identical for all the points of irreducible Brillouin zone at bounding BG edges, see Figs. 2 and 3. At a particular band, the deformation mechanism of unit cell structure for both *local* and *global* modes are identical at all points of irreducible Brillouin zone. This ensure the workability of BG in all three-directions. Besides, the proposed metastructure prototypes are easily manufacturable. In fact, a commonly available 3-D printer can be used to print the proposed metastructures. In this study, 3-D printer OBJET30 *Strata sys Ltd.* is used for 3-D printing of the proposed prototypes.

## Frequency response spectrum

**Figure 4:**
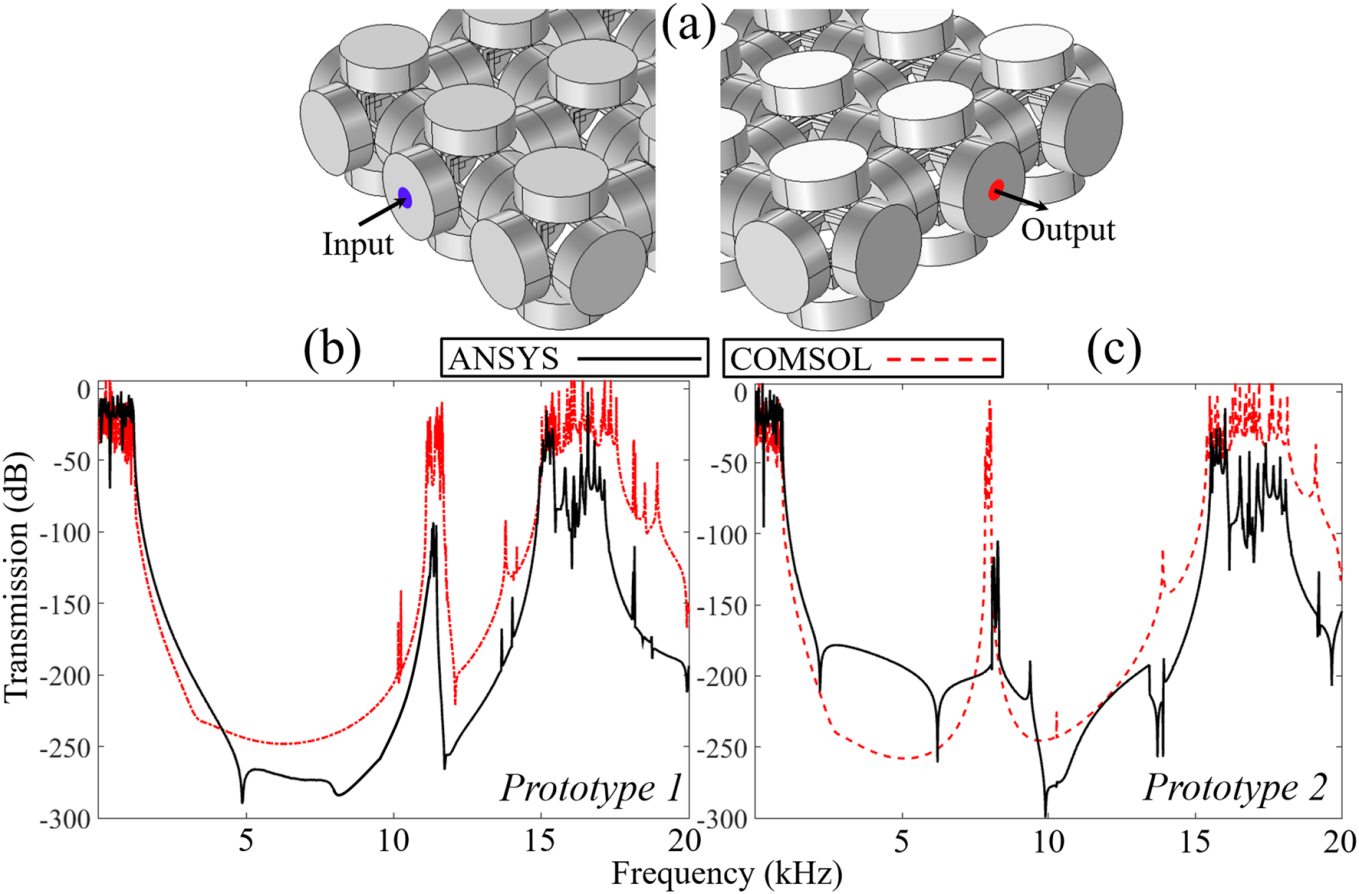
(**a**) Finite supercell with input (blue) and output (red) probes; (**b**,**c**) response spectrum for *Prototype 1* and *Prototype 2* obtained from COMSOL Multiphysics (red dashed line) and ANSYS workbench (black solid line).

The band structure presented above is obtained from COMSOL Multiphysics structure mechanics module where the Floquet–Bloch periodicity condition is applied on all the edges of cylindrical masses that made the structure infinitely periodic in the *x*–*y*–*z* directions. Some reported studies^15,19,20,32^ indicate one possible way to visualize the vibration mitigation capability from the proposed metastructures is to build a finite array of unit cell structures and to perform a frequency response study. In this regard, a 3 × 3 × 1 supercell structure is constructed and frequency response study by two different finite element codes COMSOL Multiphysics and ANSYS workbench is performed. Two FEA codes are adopted to double check the accuracy of numerical results. As shown in Fig. 4a, a harmonic excitation force is applied at the left-edge and the response in the form of displacement is record at the right-edge. The input and output displacement fields are recorded with the help of probes and the transmission ratio $T = 20\log_{10} (u_{{{\text{out}}}} /u_{{{\text{in}}}} )$ is calculated.

**Figure 5:**
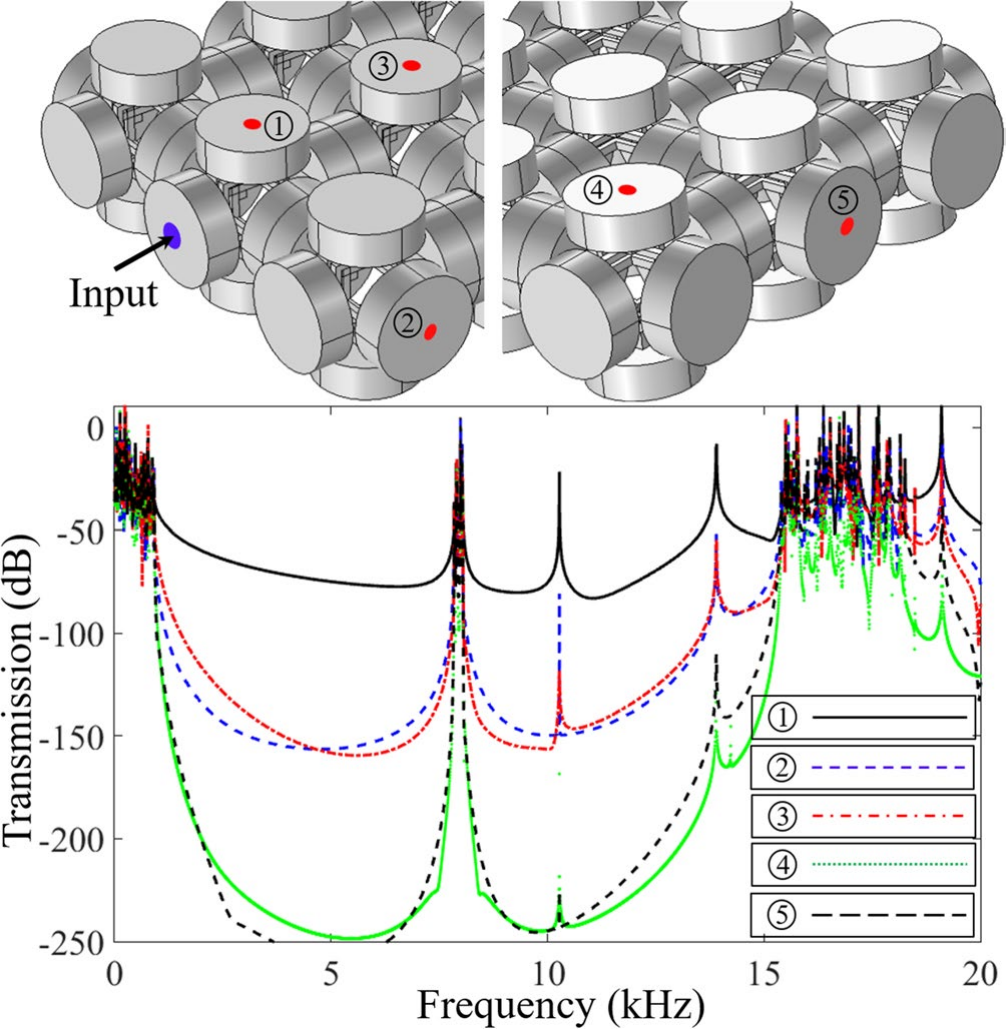
*Prototype 2:* displacement fields recorded at various probe locations numbered from 1 to 5. The BG width is independent of probe location while the attenuation depth increases if the point probe is away from the excitation source.

The wave transmission curves obtained by FE numerical simulations are presented in Fig. 4b,c that correspond to the $\Gamma - X$ direction of the irreducible Brillouin zone. Since BGs are uniformly distributed in all three directions of the Brillouin zone, thus the wave propagation in any direction leads to an identical response spectrum. From the frequency response spectrum shown in Fig. 4b,c one can observe the presence of ultrawide BG for both metastructure prototypes. Identical to the band structures presented in Figs. 2 and 3, the frequency response spectrum reveals the presence of BGs from 1247 to 11,319 Hz and from 12,044 to 14,021 Hz for *Prototype* 1, and for *Prototype* 2 the wave attenuation zones are noted that start from 929 to 7847 Hz and 8065 to 14,253 Hz. Inside the BG frequency region one can physically visualize the vibration attenuation capability from the proposed monolithic metastructures. Furthermore, the intensity of wave attenuation is studied by placing a point probe at different locations of the finite array model. As shown in Fig. 5, different probe positions on *Prototype* 2 are marked to investigate the influence of BG width and attenuation depth on the frequency response spectrum. It is found that, the BG width remains persistent for all the cases while the attenuation depth varies. For probe 1 the wave attenuation is around − 50 dB while for probe 2–3 being equidistance, have identical attenuation depth of around − 150 dB. Similarly, probe 4–5 have attenuation depth of approximately − 250 dB. In conclusion, an increase in the number of unit cell structure robustly attenuate the propagating elastic waves inside the BG frequencies.

**Figure 6:**
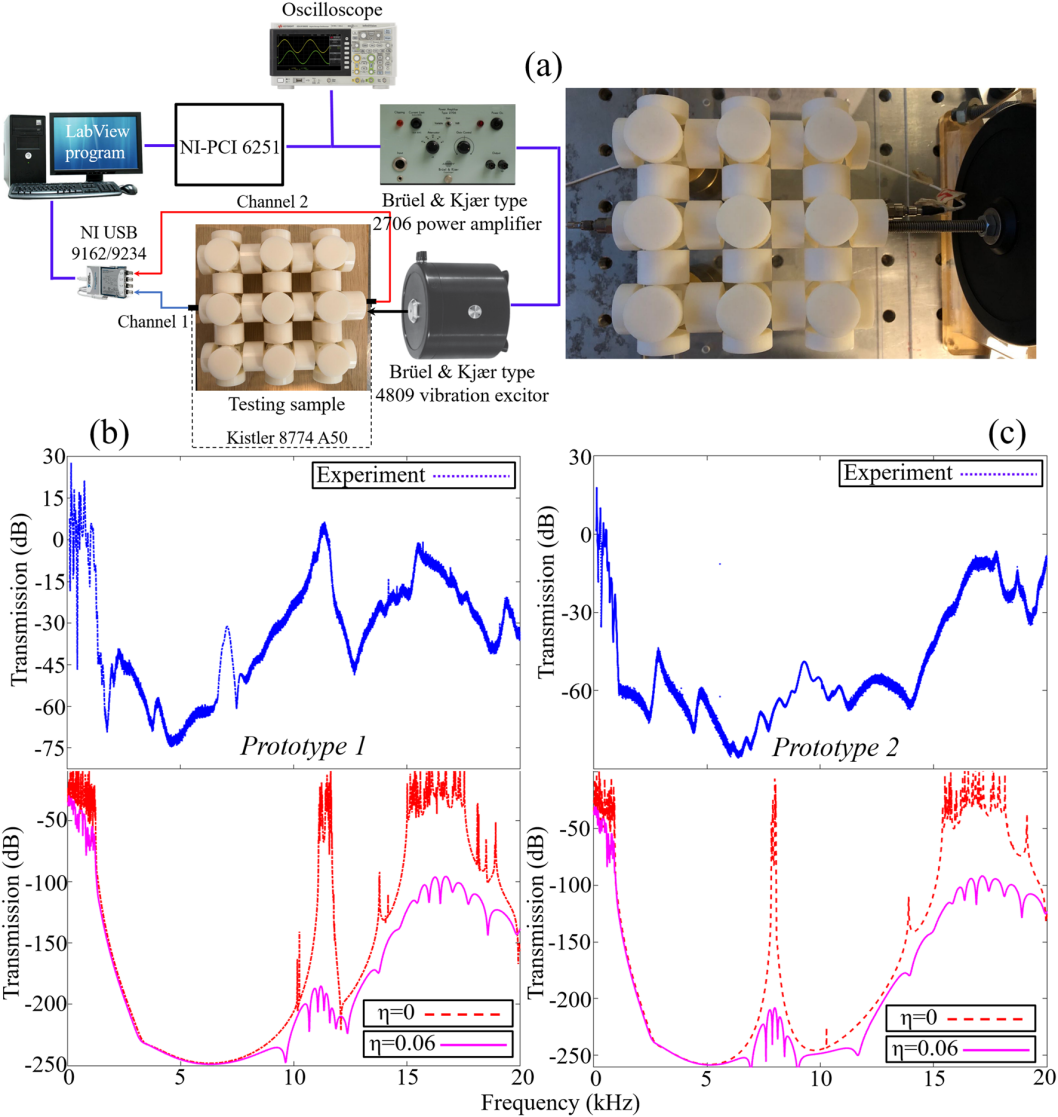
(**a**) Experiment setup; (**b**,**c**) *Prototype 1* and *Prototype 2* experiment and numerical result. For numerical transmission curve, the effect of material loss factor is $\eta = 0.06$. Excellent agreement between numerical and experiment result is obtained.

The numerical response spectrum reported in Figs. 4 and 5 do not take into consideration the effect of material damping. Usually polymeric materials possess high material damping, thus the consideration of material loss factor $\eta$ on response spectrum is of high interest. The dynamic mechanical analysis (DMA) test is performed on VeroWhite specimen to determine the material loss factor $\eta$^15^ and this parameter is incorporated in the FEA codes to investigate the effect of material losses on the wave transmission curves. For VeroWhite, the material loss factor corresponding to 20 °C (room temperature) is $\eta = 0.06$. Further details about DMA test is given in the Supplementary materials. As shown in Fig. 6, the material damping tends to flatten the wave transmission peaks and spread the vibration attenuation beyond the closing bounding edge of the BGs. Another reason for considering the effect of material loss factor/material damping on the response spectrum is to compare the numerical solutions with experimental results.

Thanks to additive manufacturing technology, by using 3-D printer OBJET30 *Strata sys Ltd* both prototypes are constructed and a low amplitude vibration test is performed to investigate the real time vibration attenuation characteristics from both metastructures. In Fig. 6a, the experiment setup and details are presented^28^ while Fig. 6b,c compare the numerical and experiment wave transmission curves for both prototypes. A good agreement in term of vibration attenuation between the numerical and experimental results is obtained. One can observe identical attenuation bandwidths for both numerical and experimental wave transmission curves however due to accelerometers limited precision compare to both FEA codes, there is a discrepancy between numerical and experiment wave attenuation depth. Other minor discrepancies between numerical and experimental results could be caused by manufacturing imperfections, material anisotropy, dimensional fidelity, surface roughness and experimental equipment limitations. For a detailed study on impact of manufacturing processes on acoustic metamaterial performance, one can refer to recent works by John Kennedy and co-workers^33,34^. Thus, it can be assumed that 3-D printing process could be one of the possible reasons for the discrepancies between numerical and experimental results. Because of the inherent material damping, one can observe the narrow passband observed around 8000 Hz in the wave transmission curve of *Prototype 2* vanishes and it spreads the wave attenuation zone over broadband frequency range. Besides, the polymeric material in numerical modelling is assumed homogenous, linear elastic and isotropic. However, this assumption may be too approximate for polymeric materials used in the 3-D printing with a higher material losses and anisotropic material properties. Hence, this could be another possible reason for minor discrepancy between numerical and experimental results.

## Experiment setup

The experiment setup is illustrated in Fig. 6a. ^28^ A vibration excitor (Brüel & Kjær type 4809 vibration excitor) is used as an actuator to transmit sine waves of varying frequencies. The sine waves are generated by NI PCI 6251 and are amplified with a Brüel & Kjær type 2706 power amplifier. A sine-sweep vibration testing approach is adopted where sine waves are swept from 100 to 20,000 Hz. By drilling small holes at both ends of the sample, the vibration excitor nob and two accelerometers (Kistler 8774 A50 with sensitivity 100 mV/g) are mounted. The input and output acceleration data are acquired by data acquisition module NI USB 9162 and 24 bit NI 9234. The result obtained is postprocessed by a computer system that is connected with data acquisition module and built-in LabView program. The response spectrum is calculated by $T\,\left( {{\text{dB}}} \right) = 20\log_{10} (a_{{{\text{out}}}} /a_{{{\text{in}}}} )$ where $a_{{{\text{out}}}}$, $a_{{{\text{in}}}}$ are the output and input acceleration quantities obtained from the output and input accelerometers, respectively.

## Method

For unit cell structure shown in Fig. 1a, the Floquet–Bloch periodicity condition is applied on all sides of the sphere in all three-directions (six boundaries) and the wavenumber is swept across the boundary of the irreducible Brillouin zone (IBZ) to obtain the band structures. A monoatomic mass-spring chain is developed to calculate the acoustic mode frequency and we compare this frequency with numerically obtained BG opening frequency that is *global* resonant mode. Numerical simulation is conducted by two different FEA codes, COMSOL Multiphysics 5.4 and ANSYS R1 2020 to double check the accuracy of numerical results. The band structures are obtained from COMSOL Multiphysics due to the flexibility in applying Floquet–Bloch periodicity condition. Subsequently a frequency response study is performed on finite array model by both FEA codes to visualize the vibration attenuation inside the BG frequencies. Both FEA codes yield very agreeable response spectra and wave attenuation zone. Finally, to corroborate the numerical findings, the 3-D prototypes are developed by using 3-D printing technology OBJET60 *Strata Sys Ltd* and low amplitude vibration test is conducted to validate the numerical findings. Throughout the study, a good agreement between theoretical, numerical and experimental results is observed.

## Conclusion

This study proposes two types of phononic metastructures prototypes that govern extremely wide low frequency bandgap for vibration and noise control. The proposed phononic metastructures facilitate all-directional wave control provided that the frequency of propagating wave lies inside the bandgap frequency range. The study is conducted by two finite element numerical models. The numerical results are compared and validated by an analytical model and by performing experimental vibration tests on the 3-D printed prototypes. Initially, an analytical model based on a monoatomic mass-spring chain is established to validate the acoustic mode frequency responsible for opening of the BG. The numerical model for wave dispersion study is also developed to obtain the band structures and to highlight the ultrawide bandgaps. Through numerical modal analysis, the *local* and *global* resonant modes associated to the bandgap closing and opening bounding edges, respectively, are also demonstrated and discussed. By principle of mode separation or modal masses participation, the ultrawide bandgap generation mechanism is elaborated. The significant differences between the *global* and *local* resonant modes caused by the proper engineering design of metastructures are found to result in the birth of ultrawide bandgaps. To envisage the wave attenuation inside the bandgap frequencies, a finite supercell structure is created and a numerical frequency response study by two different commercial finite element models is performed. The frequency response spectrum by both numerical approaches demonstrate robust wave attenuation inside the bandgap frequencies. By additive manufacturing, 3-D prototypes are printed and vibration tests are performed to further corroborate our numerical findings. Both numerical simulation and experimental results show good agreement. In the light of this study, the proposed monolithic metastructure designs are likely to have potential applications in vibration absorption facilities to attenuate vibration and noises at a wide frequency spectrum. The monolithic design and the proposed structural configurations make the fabrication and manufacturing works easier. Such innovative metastructure designs can be of prime interest for both elastic waves manipulation and underwater acoustic applications where all-directional wave control is desirable.

## Supplementary Information

Supplementary Information.

Supplementary Figure S1.

Supplementary Figure S2.

Supplementary Figure S3a–c.

Supplementary Figure S3d.

Supplementary Figure S4.

Supplementary Figure S5a–c.

Supplementary Figure S5d.

Supplementary Figure S6.

Supplementary Figure S7a–c.

Supplementary Figure S7d.

Supplementary Figure S8.

Supplementary Figure S9a–c.

Supplementary Figure S9d.

Supplementary Figure S10.

Supplementary Figure S11.
