# Supplementary material for: Phononic metastructures with ultrawide low frequency three-dimensional bandgaps as broadband low frequency filter
Source: Sci Rep. 2021 Mar 30;11:7137. doi: 10.1038/s41598-021-86520-8 (PMC8010083; doi:10.1038/s41598-021-86520-8)
Supplement: Supplementary file 1 — Supplementary Information. [file 41598_2021_86520_MOESM1_ESM.docx]

**Phononic Metastructures with Ultrawide Low Frequency Three-dimensional Bandgaps as Broadband Low Frequency Filter**

Muhammad^1,2^ and C.W. Lim^1,2^,*

^1^City University of Hong Kong Shenzhen Research Institute, Shenzhen, P.R. China

^2^Department of Architecture and Civil Engineering, City University of Hong Kong, Kowloon, Hong Kong SAR, P.R. China

*Corresponding author, email: [bccwlim@cityu.edu.hk](mailto:bccwlim@cityu.edu.hk)

**Supplementary Material**

**Analytical model**

Consider a unit cell of a monatomic mass-spring system indicated by a black frame as shown in Fig. S1, with a rigid mass $m$, a linear spring with stiffness $k$, and $L$ is the lattice constant.


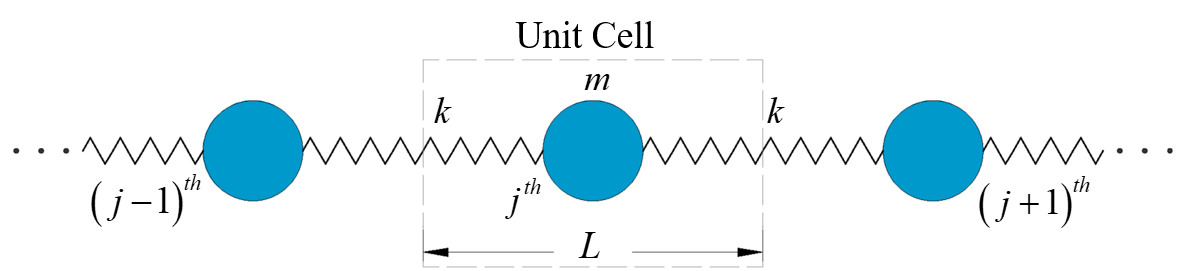


Fig. S1. A simple periodic mass-spring chain system

By applying Newton’s second law of motion to this system and in the absence of an external force, the governing equation of motion for the $j^{th}$ unit cell can be written as

(S1)

where $u_{j}$ is the displacement of the $j^{th}$ unit cell. Assuming a harmonic solution of this linear system given as

$u_{j}=Ae^{ij\kappa}e^{-i\omega t}$ (S2)

where $A$ is the amplitude, $\kappa$ is the wave number and $i=\sqrt{-1}$ is the imaginary unit. By substituting Eq. (S2) into Eq. (S1), the equation of motion becomes

$\left[-\omega^{2}m+k\left(2-e^{-i\kappa}-e^{i\kappa}\right)\right]Ae^{ij\kappa}e^{-i\omega t}=0$ (S3)

For a nontrivial solution of Eq. (S3) where the amplitude $A\neq0$, we have the determinant of Eq. (3) being zero, which gives

$\left|-\omega^{2}m+k\left(2-e^{-i\kappa}-e^{i\kappa}\right)\right|=0$ (S4)

Thus, using Euler's formula $e^{\pm i\kappa}=\cos\kappa\pm i\sin\kappa$, the frequency $\omega$ is obtained as

$\omega^{2}=\frac{2\left(1-\cos\kappa\right)k}{m}$ (S5)

It is known that the natural frequency of the monatomic lattice system can be expressed as ^1^

$\omega_{0}=\sqrt{k/m}$ (S6)

Thus, the dimensionless frequency $\Omega=\omega/\omega_{0}$ is given by

$\Omega^{2}=2\left(1-\cos\kappa\right)$ (S7)

where for a positive value of $\Omega^{2}$ and a wave with an arbitrary wavenumber $\kappa$, $\left(1-\cos\kappa\right)$ is limited within the range $\left[0,2\right]$, that leads to the limit for the dimensionless frequency as $\Omega\in\left[0,2\right]$. Thus, the frequency of wave which is permitted to propagate in this structure cannot exceed $2\omega_{0}$.


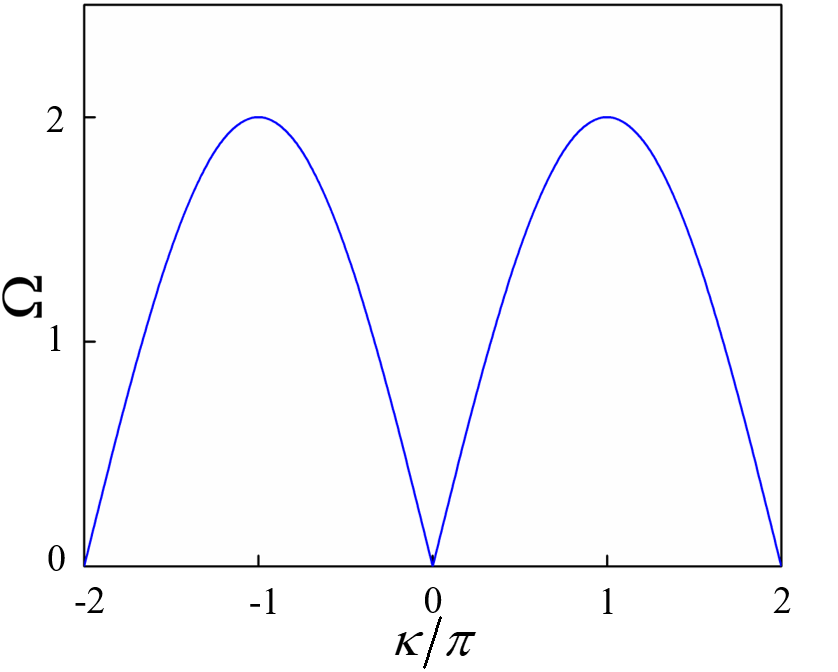


Fig. S2. Dispersion relation for the monoatomic mass-spring system by sweeping wavenumber $\kappa$ from $-2\pi$ to $2\pi$.

The dispersion relation is shown in Fig. S2. It is observed that the dispersion relation is periodic with period $2\pi$ and a stop band occurs when $\Omega>2$ with a passband in the range $\Omega\in\left[0,2\right]$. Further details can be found in Hussein et al.^2,3^.

**Basic theory and mathematical framework**

For a perfectly elastic, homogenous and isotropic system, the governing equation for in-plane wave propagation can be expressed as^21^

$\nabla\cdot\left[\mathbf{C}\left(\mathbf{r}\right):\nabla\mathbf{u}\left(\mathbf{r}\right)\right]=\rho\left(\mathbf{r}\right)\frac{\partial^{2}\mathbf{u}\left(\mathbf{r}\right)}{\partial t^{2}}$ (S8)

where $\nabla=\sum_{i=x,y,z} \mathbf{e}_{i}\frac{\partial}{\partial x_{i}}$ designates the vector differential operator in which $\mathbf{e}_{i}$ is the unit base vector, $\mathbf{r}=\left(x,y,z\right)$ is position vector, $t$ is time, and $\mathbf{C}\left(\mathbf{r}\right)$, $\mathbf{u}\left(\mathbf{r}\right)$ and $\rho\left(\mathbf{r}\right)$ are position dependent fourth order elasticity tensor, displacement vector and mass density, respectively. Further, “”, “” are the vector dot product and double-dot product of two dyadics, respectively. The Floquet-Bloch periodicity condition is applied along the $xyz$ directions. According to this theory, the solution to Eq. (S8) can be represented by

$\mathbf{u}\left(\mathbf{r},t\right)=e^{i\left(\mathbf{k}\cdot\mathbf{r}-\omega t\right)}\mathbf{u}_{\mathbf{k}}\left(\mathbf{r}\right)$ (S9)

where $\mathbf{u}_{\mathbf{k}}\left(\mathbf{r}\right)$ is displacement modulation function, $\omega$ is angular frequency and $\mathbf{k}=\left(k_{x},k_{y},k_{z}\right)$ is wavevector while $k_{x},k_{y},k_{z}$ are the wavenumbers in the $x-y-z$ directions of the Brillouin reciprocal space, respectively. Analytically, the Fourier space of Brillouin lattice vector is the reciprocal lattice and the base vectors of the reciprocal lattice $\mathbf{k}_{x}, \mathbf{k}_{y},\mathbf{k}_{z}$ can be expressed as^4^

$\mathbf{k}_{x}=\frac{2\pi\left(\mathbf{e}_{y}\times\mathbf{e}_{z}\right)}{\mathbf{e}_{x}\cdot\left(\mathbf{e}_{y}\times\mathbf{e}_{z}\right)}; \mathbf{k}_{y}=\frac{2\pi\left(\mathbf{e}_{x}\times\mathbf{e}_{z}\right)}{\mathbf{e}_{x}\cdot\left(\mathbf{e}_{y}\times\mathbf{e}_{z}\right)}; \mathbf{k}_{z}=\frac{2\pi\left(\mathbf{e}_{x}\times\mathbf{e}_{y}\right)}{\mathbf{e}_{x}\cdot\left(\mathbf{e}_{y}\times\mathbf{e}_{z}\right)}$ (S10)

where $\mathbf{e}_{x},\mathbf{e}_{y}$ and $\mathbf{e}_{z}$ are base vectors of the direct lattice vector space. As the system is periodic in three directions the irreducible Brillouin zone in $\Gamma XM\Gamma RXMR$ is considered. The extra $RXMR$ coordinates are responsible for the wavenumber in *z-*direction. The governing periodicity equation becomes

$\mathbf{u}\left(\mathbf{r}+\mathbf{a},t\right)=\mathbf{u}_{\mathbf{k}}\left(\mathbf{r}+\mathbf{a}\right)e^{i\left(\mathbf{k}\cdot\left(\mathbf{r}+\mathbf{a}\right)-\omega t\right)}=e^{i\mathbf{k}\cdot\mathbf{a}}\mathbf{u}_{\mathbf{k}}\left(\mathbf{r}\right)e^{i\left(\mathbf{k}\cdot\mathbf{r}-\omega t\right)}=e^{i\mathbf{k}\cdot\mathbf{a}}\mathbf{u}\left(\mathbf{r},t\right)$ (S11)

Further simplification of the equations leads to an eigenvalue equation of the form

$\left(\mathbf{\Phi}\left(\mathbf{k}\right)-\omega^{2}\mathbf{M}\right)\cdot\mathbf{U}=0$ (S12)

where $\mathbf{\Phi}$ is a stiffness matrix that is a function of $\mathbf{k}$ and $\mathbf{M}$ is the global mass matrix. The solution of Eq. (S12) helps plot wavenumber with eigenfrequencies that results in a dispersion response. Further details can be found in Muhammad et al.^21^.

**Some preliminary metastructure designs**

Discussion on the finalized prototypes with respect to bandgap (BG) that governs the largest relative bandwidth and ultrawide BG is presented here. The procedure involved and other preliminary designs are put forward that eventually shape the final structure. Analytical result for the preliminary designs is denoted with a red dashed line. For each design, the schematic diagram along with band structure, vibration modes of the bounding BG edges and finite unit cell based frequency response studies are conducted to demonstrate the wave attenuation inside the BG frequencies. Referring to the experiment result reported in the main paper, one can assume similar findings for these proposed designs. Apart from these, the dynamic mechanical analysis (DMA) test for determining material loss factor is explained below.

A schematic diagram of the proposed structure is presented in Fig. S3(a) where rigid cylindrical masses are supported by flexible frame assembly. The geometric parameters adopted are shown at the inset. The wave dispersion diagrams with normalized and general frequency is shown in Fig. S3(b, c) where BGs are highlighted and the widest BG with a relative bandwidth 129% is reported. The BG generation mechanism and governing physical concepts are explained in the main paper. For the reader’s reference, vibration modes corresponding to the opening (*global resonant mode*) and closing (*local resonant mode*) bounding edges are shown in Fig. S3(d). It is observed that for the first widest BG, the BG opening edge has complete modal mass participation from both rigid masses and flexural stiffness of the frame assembly. However, the closing bounding edge has only local mode participation caused by the flexural stiffness of the frame structure. A detailed analysis on global and local resonant modes and their role in the birth of ultrawide BG is given in the main paper.


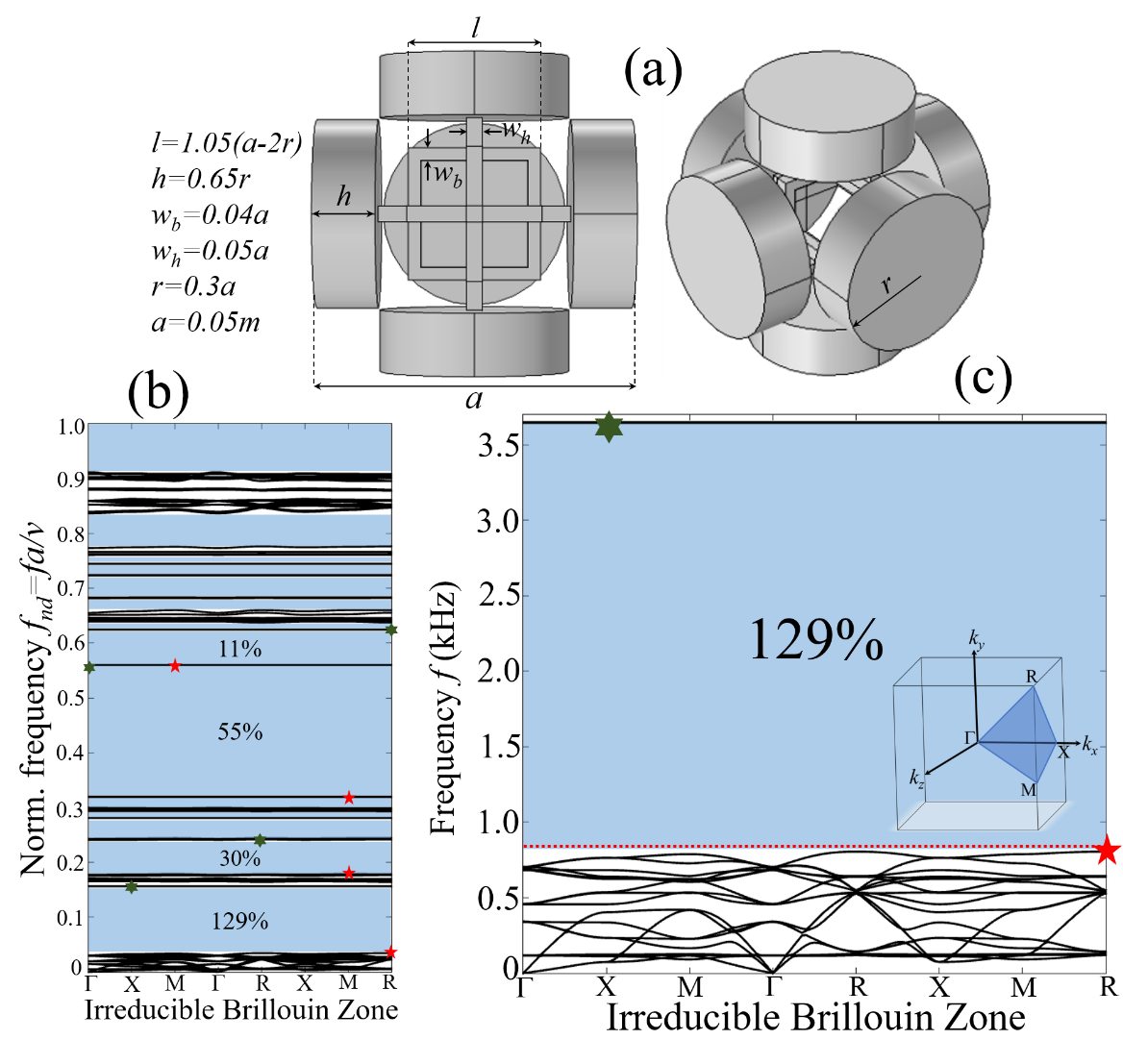

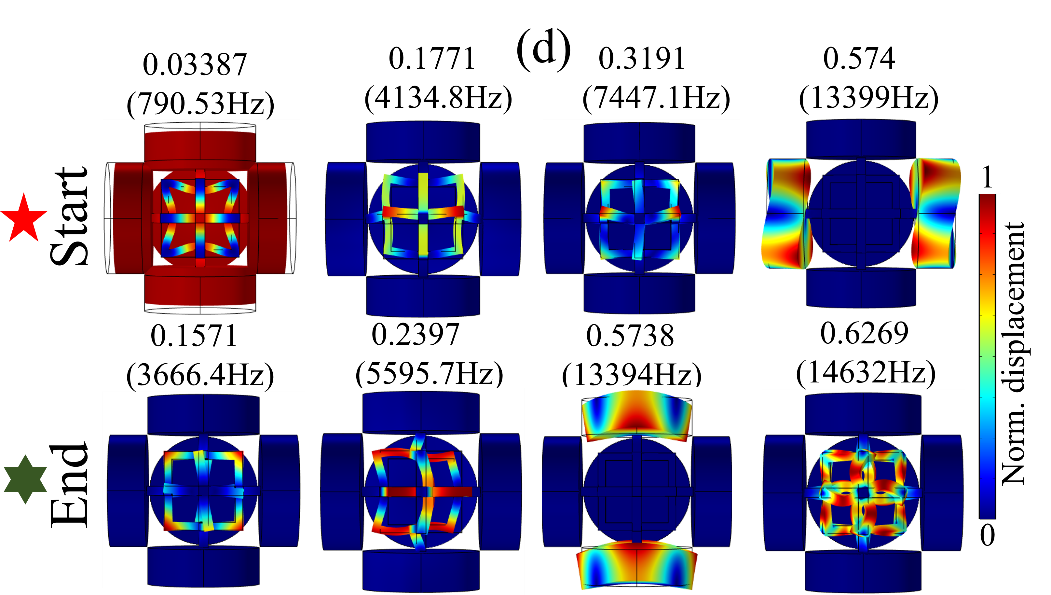


Fig. S3. (a) Proposed metastructure; (b-c) band structure with BGs for both normalized and general frequencies. The irreducible Brillouin zone is shown at the inset of Fig. S3(c). (d) Vibration modes at the bounding edges of the BGs.

To validate the wave attenuation inside the BG frequencies, a finite array of unit cells that consist of 3x3x1 array is created and the frequency response study is performed to determine the response spectrum as shown in Fig. S4. Reminiscent with the main text, the response spectrum validates the BGs reported in the band structure and significant vibration attenuation is observed for the BG frequencies. Because the purpose of this supplementary information is to discuss some preliminary designs, thus an emphasis on the COMSOL Multiphysics simulation result is presented. One can assume similar findings from FEA code ANSYS and experiment work as explained in the main text.


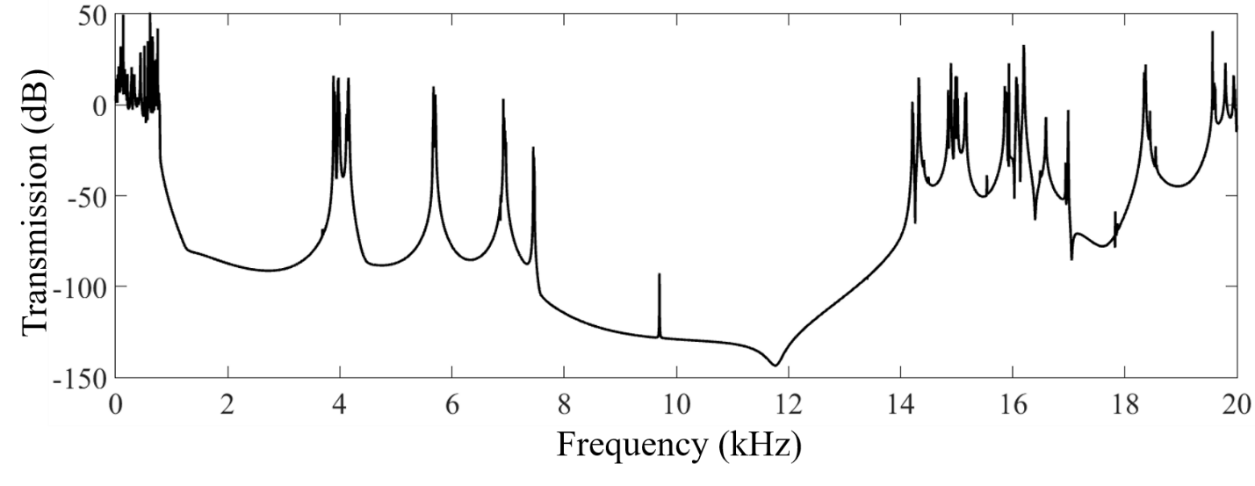


Fig. S4. Frequency response spectrum for the finite supercell design shown in Fig. S3(a).

Further, we introduce a cube mass between the frame assembly and rigid cylindrical masses to make the joint stronger. The schematic structure and geometric parameters are shown in Fig. S5(a). Similarly, the wave dispersion curve with BGs in both normalized and general frequency format is shown in Fig. S5 (b, c). The introduction of the cube masses enhances the BG width and a wider first BG with relative bandwidth 130% is obtained. The vibration modes that correspond to the bounding BG edges are shown in Fig. S5 (d). The results are identical to that explained in the main text.


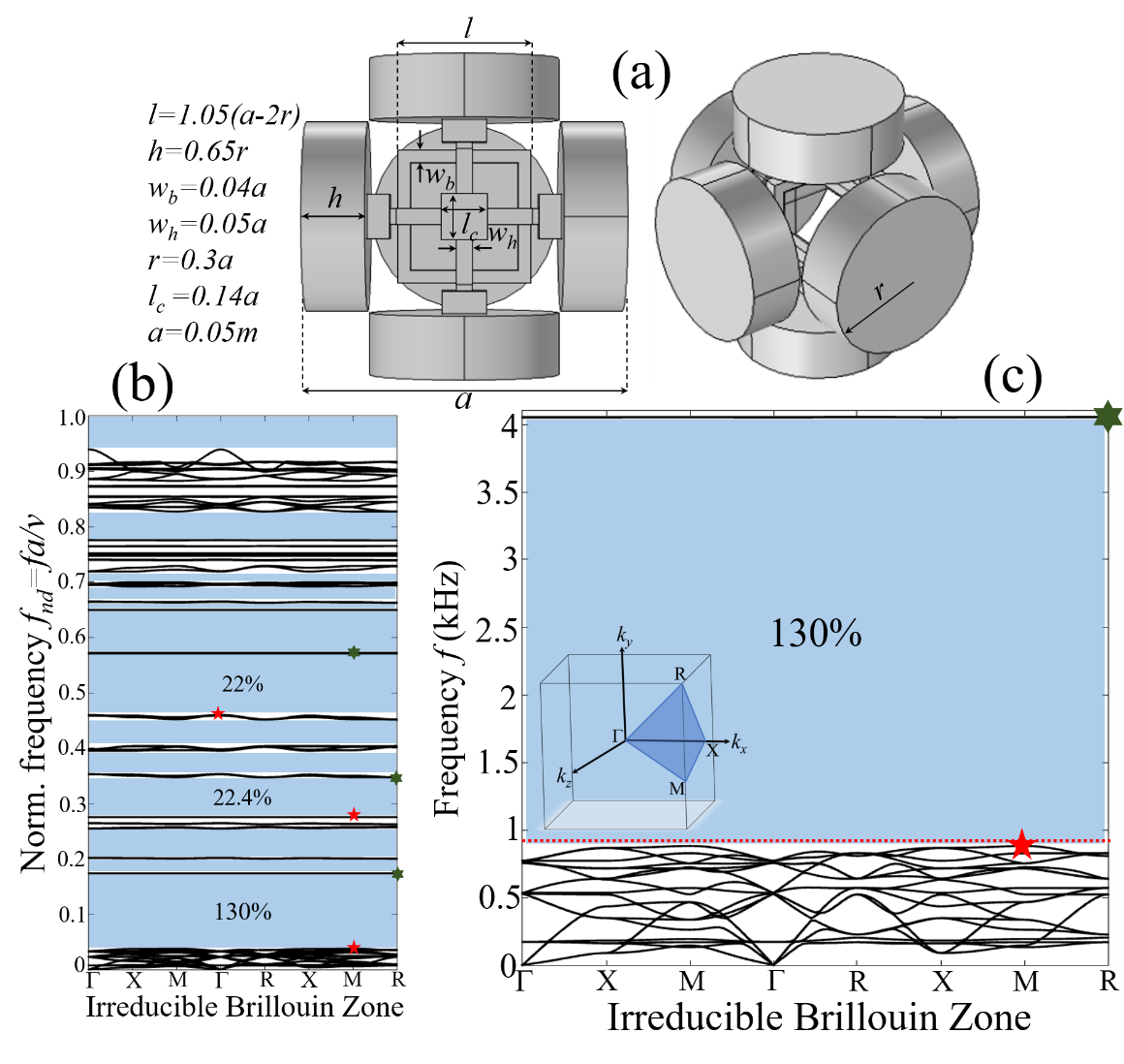

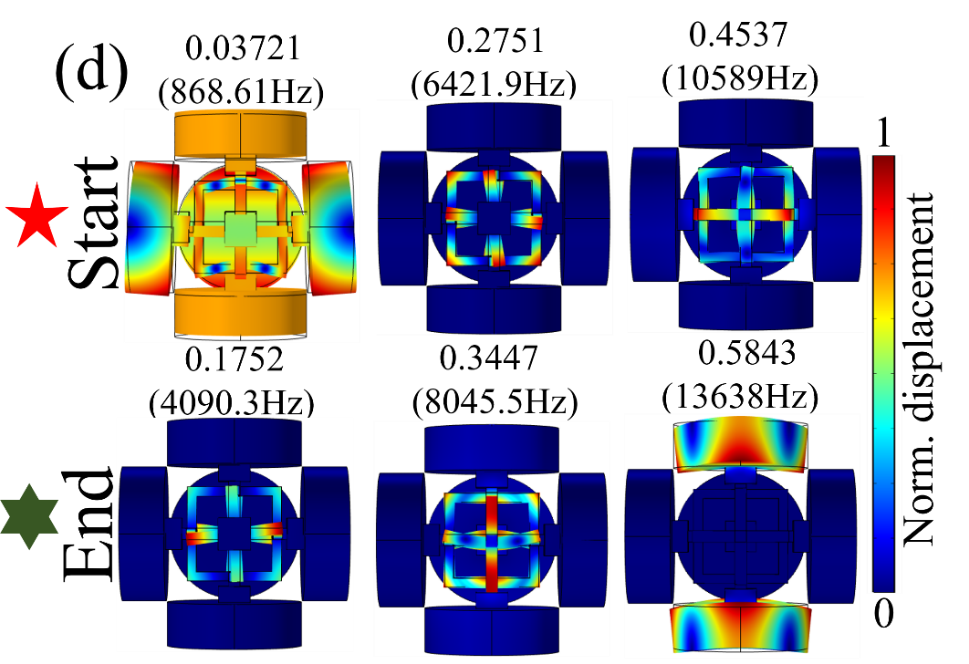


Fig. S5. (a) Schematic diagram for the proposed metastructure; (b-c) band structure with BGs for both normalized and general frequencies. The irreducible Brillouin zone is shown at the inset of Fig. S5(c). (d) Vibration modes at the bounding BG edges.

Similarly, we constructed an array of 3x3x1 supercell structure where a frequency response study is conducted. The frequency response spectrum with BGs that validates the band structure finding is reported in Fig. S6.


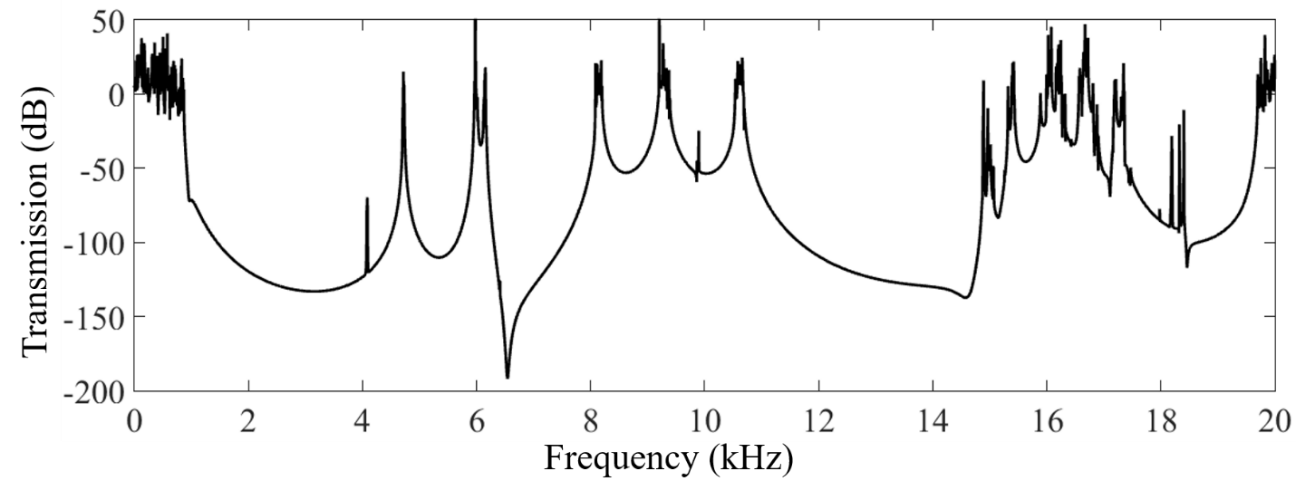


Fig. S6. Frequency response spectrum for the finite supercell structure shown in Fig. S5(a).

The prior two models have identical geometric parameters except that in the latter a cube is embedded between the supporting frame assembly and the cylindrical mass. Here, we reduce the geometric parameter for frame assembly in order to check the effect on the band structure and BGs. The details of geometric parameters are given at the inset of Fig. S7(a). The band structure with highlighted BGs and vibration modes at the bounding BG edges are shown in Fig. S7(b-d). Compared to Fig. S3 the relative bandwidth increases to 135%. The enhancement in the relative bandwidth is due to the reduction in flexural stiffness of the frame assembly that is connected with rigid cylindrical masses. Of course, further reduction in frame thickness will result in a broader BG but one should also consider manufacturing limitations and restrictions.


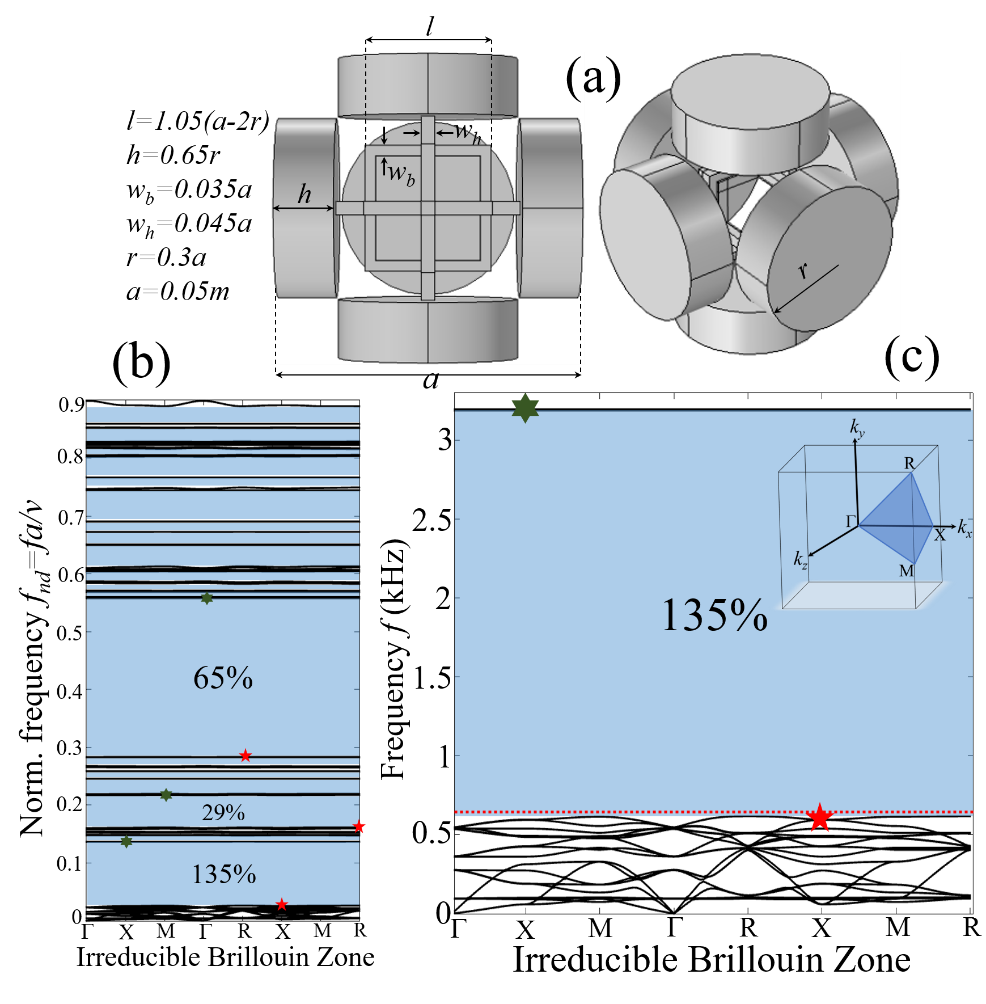


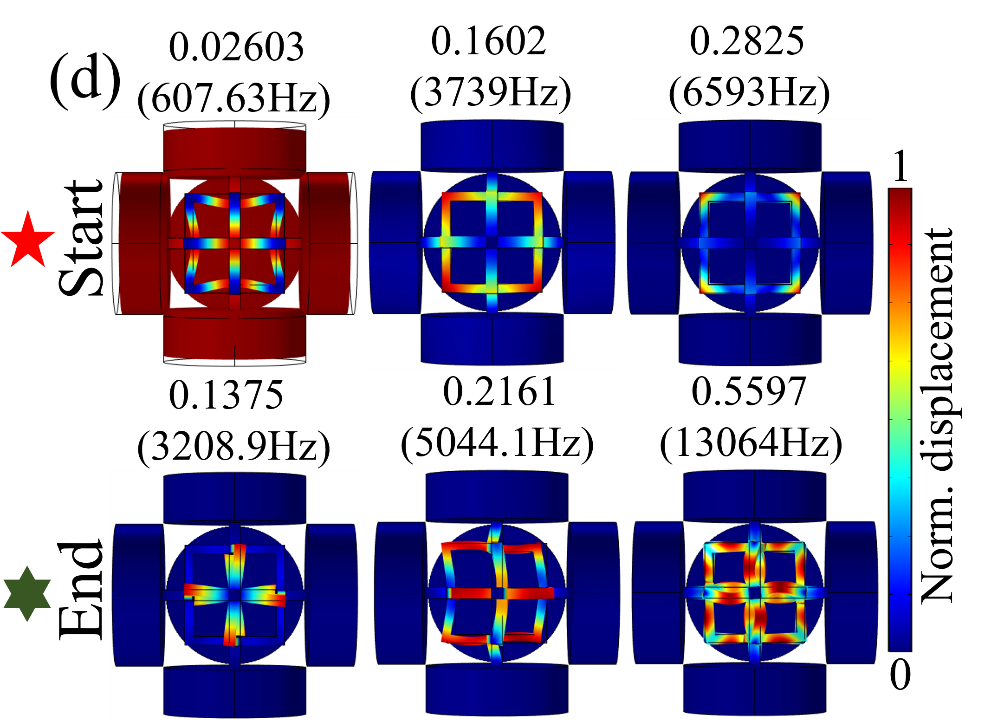


Fig. S7. (a) Schematic diagram for the proposed metastructure; (b-c) band structure with BGs with both normalized and general frequencies. The irreducible Brillouin zone is shown at the inset of Fig. S7(c). (d) Vibration modes at the bounding BG edges.

As shown in Fig. S8, a frequency response study on the 3x3x1 supercell structure is performed. An extremely wide BG identical to that highlighted in Fig. S7(b-c) is observed.


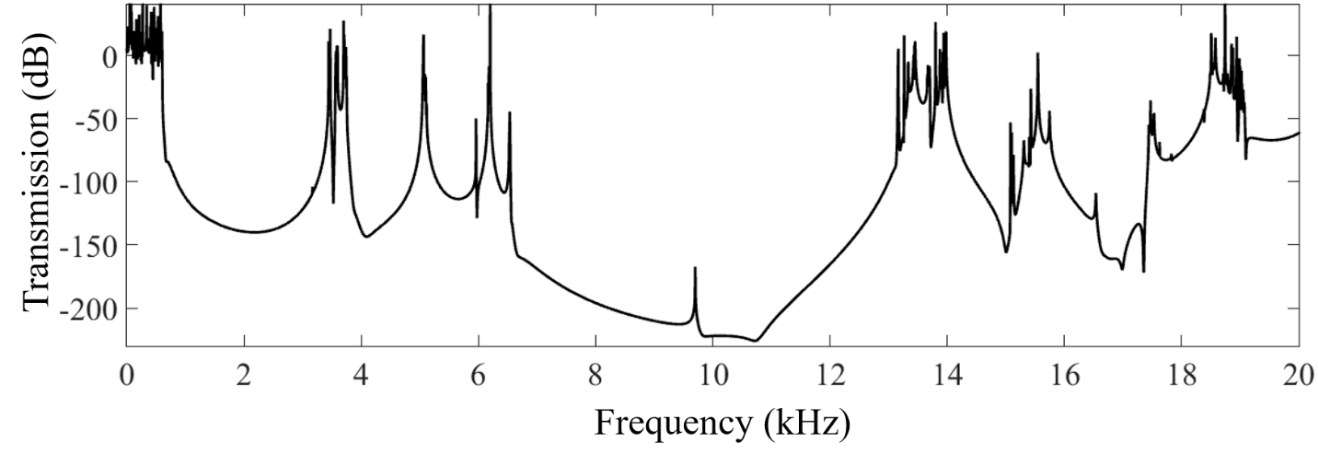


Fig. S8. Frequency response spectrum for the finite supercell design shown in Fig. S7(a).

The size of the cube is further increased as shown in Fig. S5(a) and we study its effect on the wave dispersion curve and BGs. The proposed design along with the geometric parameters is shown in Fig. S9(a). As shown in Fig. S9(b-c), an increase in the cube size helps enhance a larger participation from the modal masses that results in further widening of the BG. The relative bandwidth increases from 130%, as seen in Fig. S5(b-c), to 148.3%. The vibration modes that correspond to the bounding BG edges are shown in Fig. S9(d).


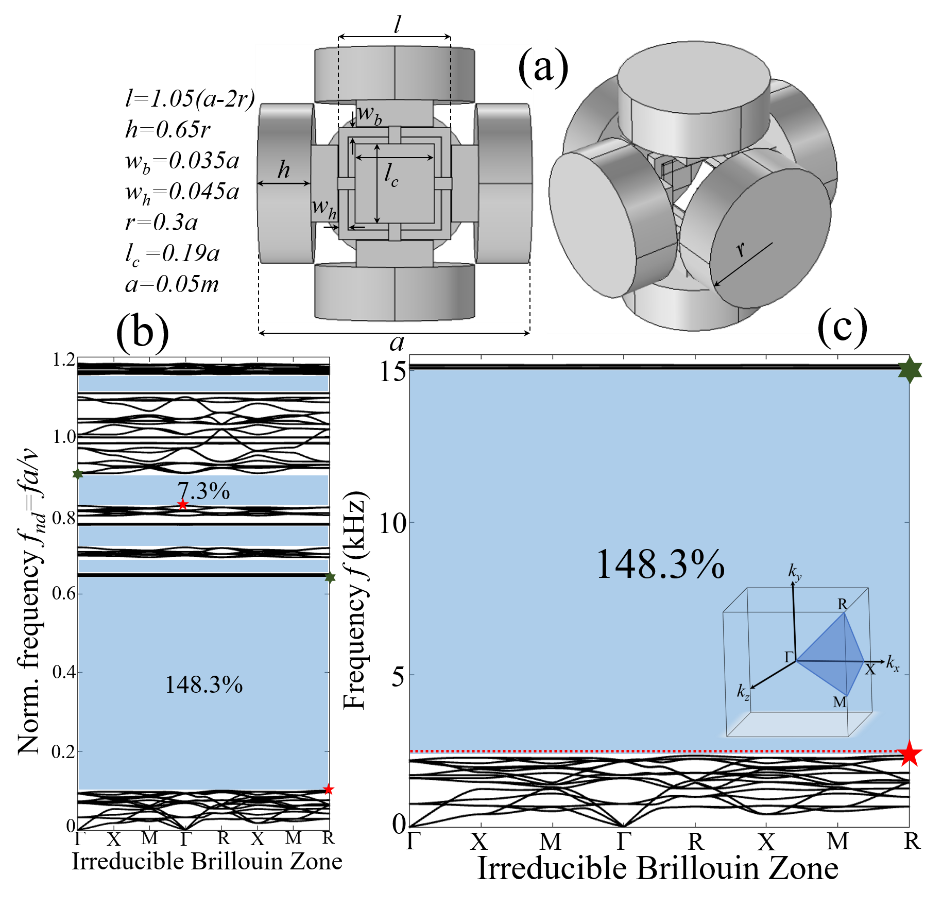


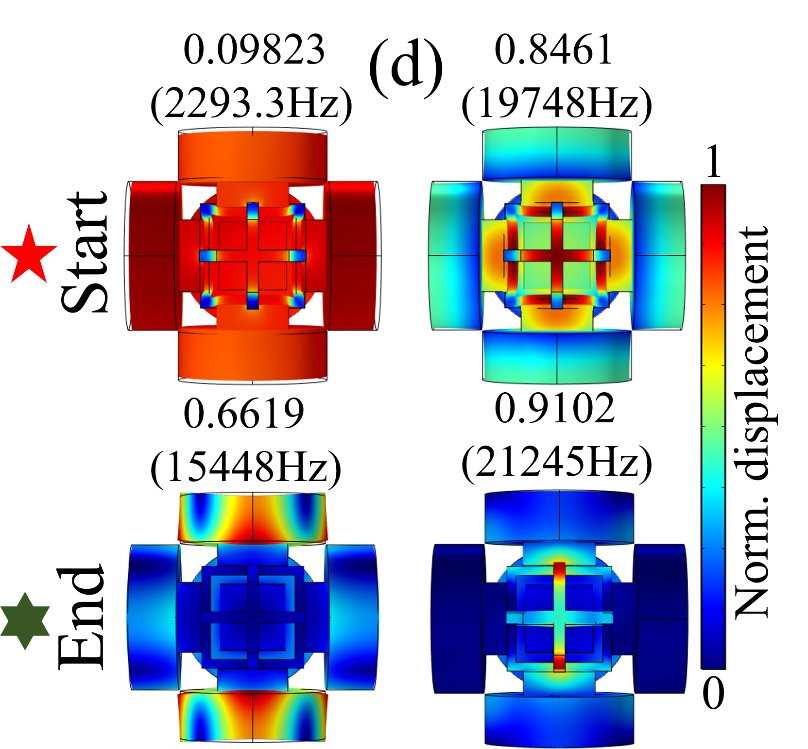


Fig. S9. (a) Schematic diagram for the proposed metastructure; (b-c) band structure with BGs for both normalized and general frequencies. The irreducible Brillouin zone is shown at the inset of Fig. S9(c). (d) Vibration modes at the bounding BG edges.

Similarly, an array of 3x3x1 supercell structure is tested for the frequency response study to visualize the vibration attenuation properties. From Fig. S10, vibration attenuation in the BG frequency regions is observed.


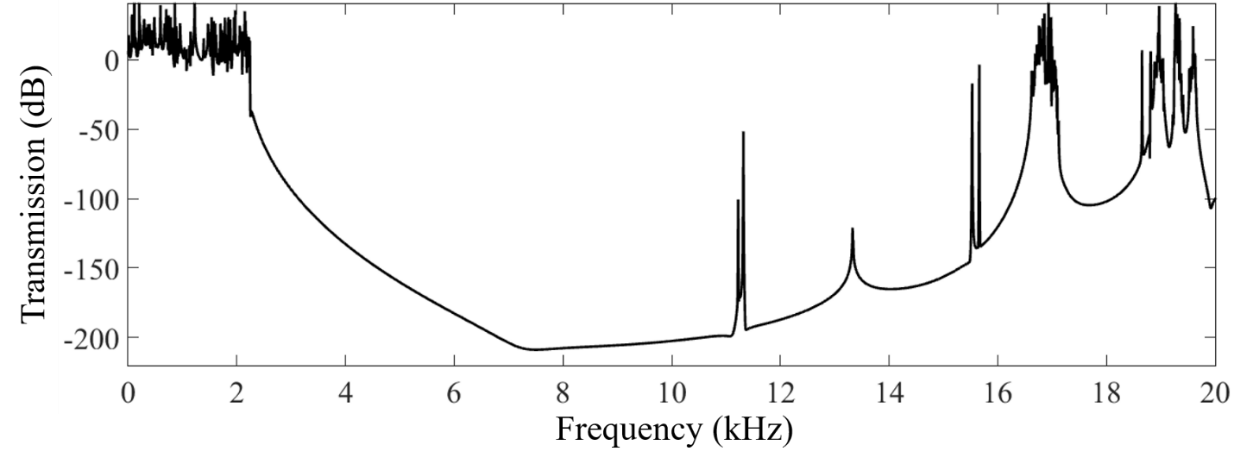


Fig. S10. Frequency response spectrum for the finite supercell design shown in Fig. S9(a).

**Dynamic mechanical analysis (DMA) tests**

Dynamical mechanical analysis (DMA) test is an effective testing technique that is widely used to characterize material properties w.r.t frequency, time, temperature, atmosphere, stress etc. A sinusoidal deformation is transmitted from a shaft on a known geometric-sized specimen. Either the stress or strain is kept constant and the corresponding storage and loss modulus of material at varying temperature is determined. The frequency is kept constant and/or varied and the force is determined by stress-strain relationship. DMA measures stiffness and damping properties and they are presented as modulus and tan$\delta$. The $\tan\delta$ is a parameter for defining material damping. Because a sinusoidal force is applied, the modulus is expressed in the form of in-phase (storage modulus) and out-of-phase (loss modulus) components. The ratio of storage to loss modulus is a measure of energy dissipation per-cycle or $\tan\delta$/damping. This parameter shows how well a material dissipate energy under cyclic loading as tangent of phase angle. In addition, this parameter varies with the state of material, temperature and frequency. In this study, the strain and frequency are kept constant and the loss and storage moduli at varying temperature are determined. The testing details are as follow.

The experiment setup is shown in Fig. S11(a) where a rectangular specimen of 35x15x5 mm in size is mounted in a testing chamber of DMA Q800 V21.2 machine. The strain/deformation is kept at $10\mu m$ and a cyclic load of 1Hz is applied that tends to vibrate the specimen like a simply supported beam subjected to a harmonic excitation. Based on the constant strain value, the apparatus shows the application of a force of 10N-12N on the specimen at varying temperature. A good waveform is observed that indicates accurate reported result. The result is presented in Fig. S11(b, c) and $\eta=0.06$ corresponding to 20^o^C is adopted in the FEA models.


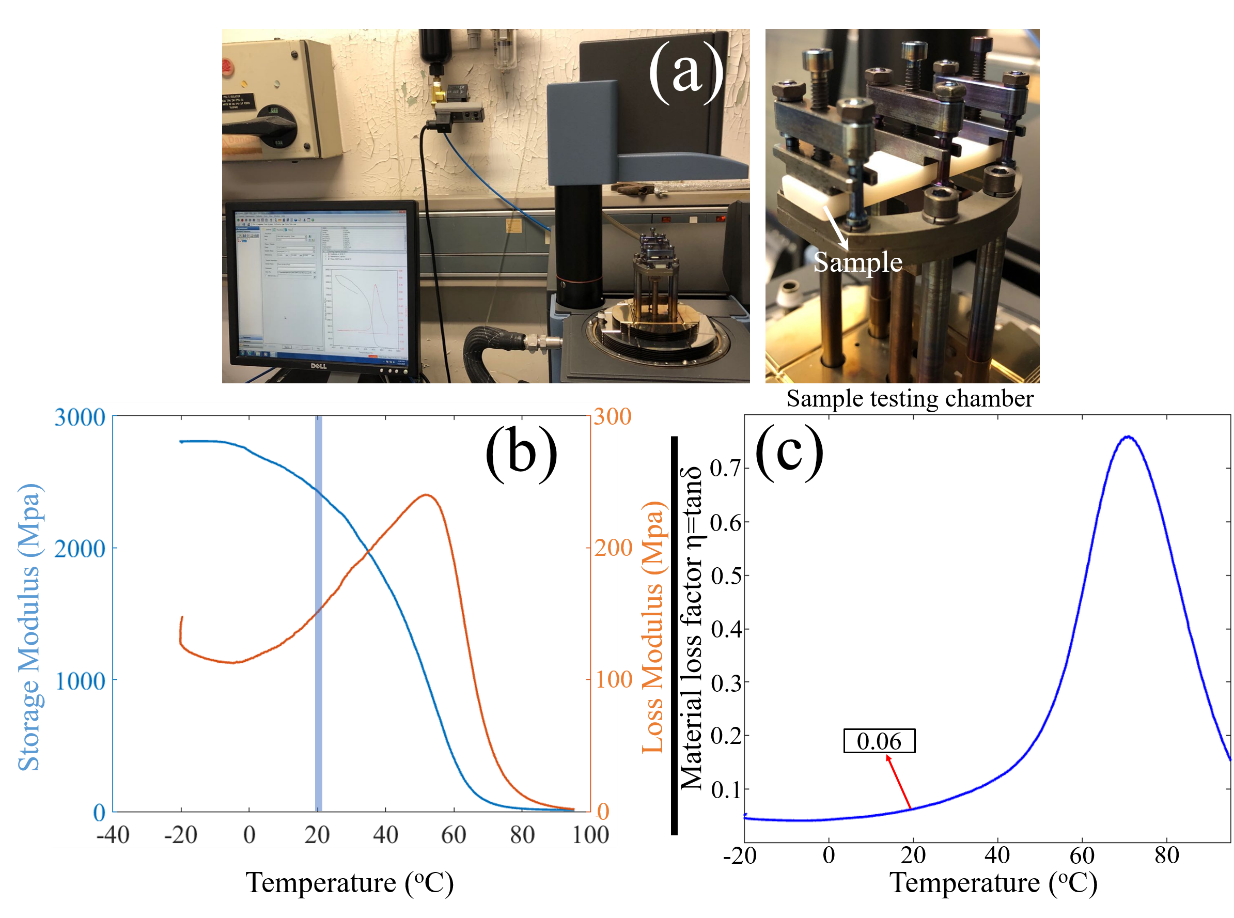


Fig. S11. (a) DMA test setup; (b) storage and loss modulus at varying temperature; (c) material loss factor $\eta$ as function of temperature. The value adopted is highlighted and marked in a box.

**References**

1 Craig Jr, R. R. & Kurdila, A. J. *Fundamentals of structural dynamics*. (John Wiley & Sons, 2006).

2 Hussein, M. I., Leamy, M. J. & Ruzzene, M. Dynamics of Phononic Materials and Structures: Historical Origins, Recent Progress, and Future Outlook. *Applied Mechanics Reviews* **66**, 040802, doi:10.1115/1.4026911 (2014).

3 Hussein, M. I. Theory of damped Bloch waves in elastic media. *Physical Review B* **80**, 1-4, doi:ARTN 21230110.1103/PhysRevB.80.212301 (2009).

4 Muhammad, Lim, C. W. & Reddy, J. N. Built-up structural steel sections as seismic metamaterials for surface wave attenuation with low frequency wide bandgap in layered soil medium. *Eng Struct* **188**, 440-451, doi:10.1016/j.engstruct.2019.03.046 (2019).
