# Supplementary figures and images for: Phononic metastructures with ultrawide low frequency three-dimensional bandgaps as broadband low frequency filter
Source: Sci Rep. 2021 Mar 30;11:7137. doi: 10.1038/s41598-021-86520-8 (PMC8010083; doi:10.1038/s41598-021-86520-8)

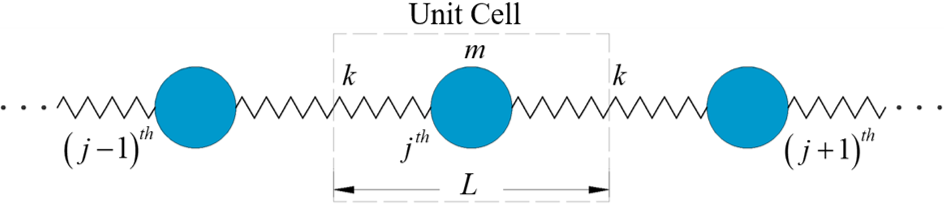

Supplement: Supplementary file 2 — Supplementary Figure S1. [file 41598_2021_86520_MOESM2_ESM.tif]

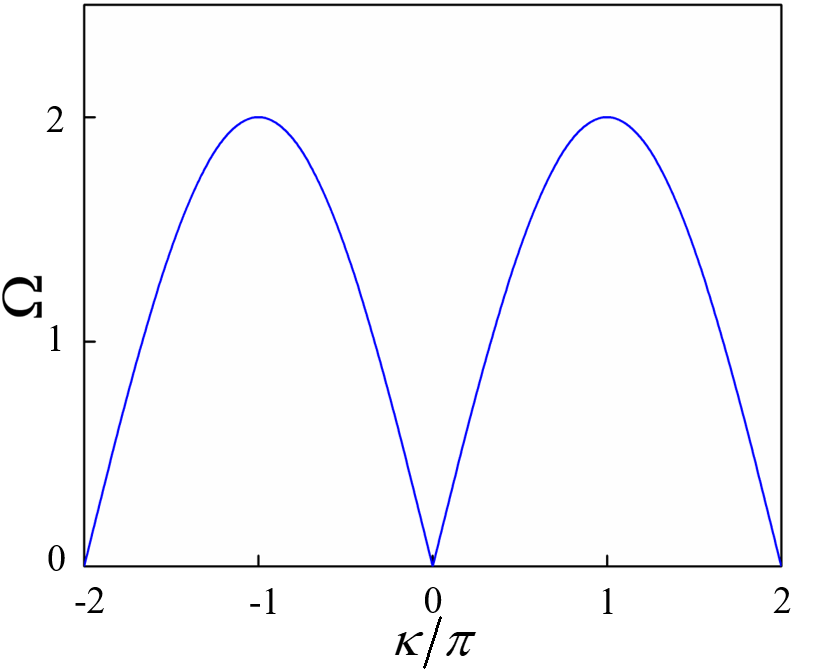

Supplement: Supplementary file 3 — Supplementary Figure S2. [file 41598_2021_86520_MOESM3_ESM.tif]

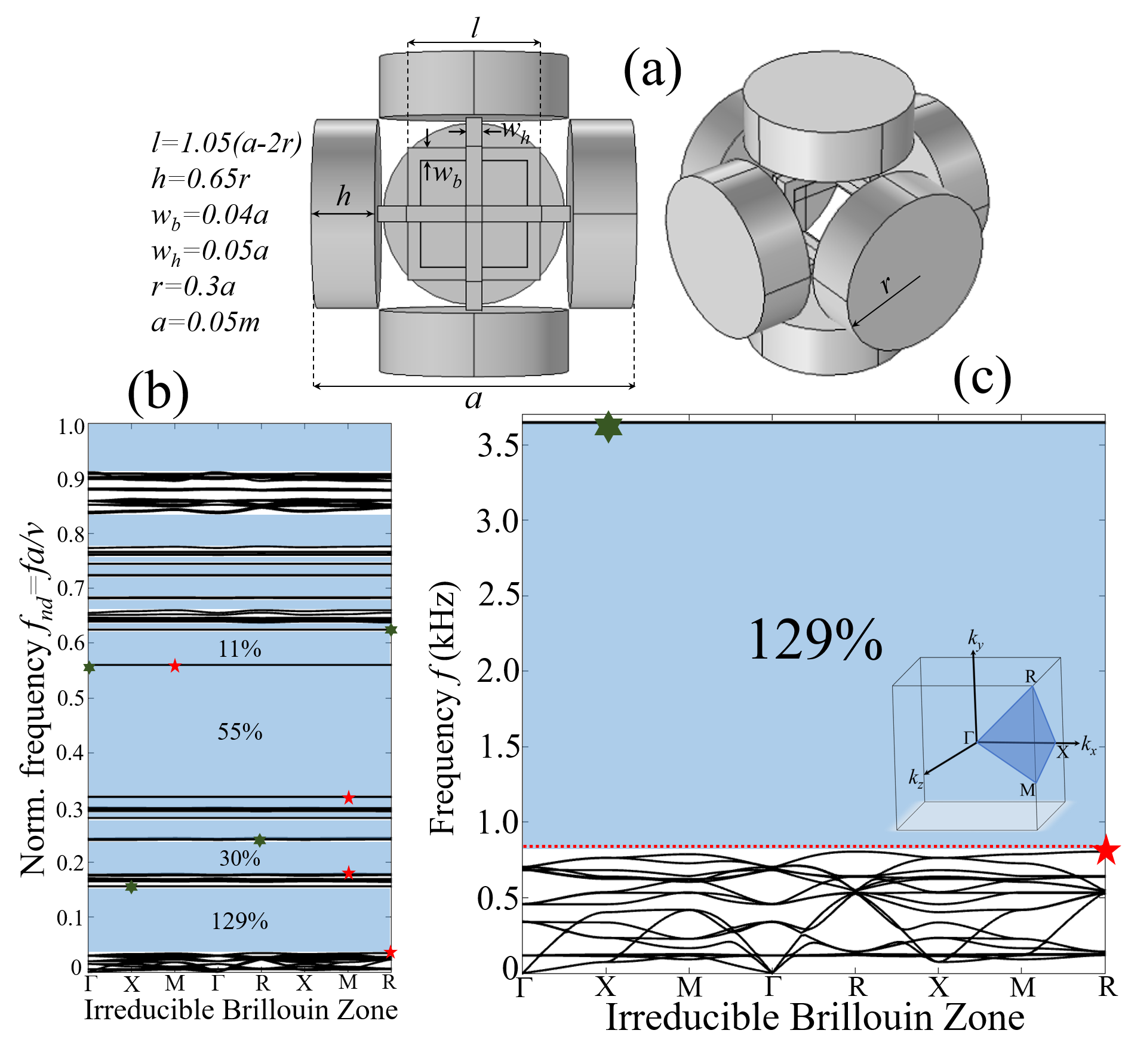

Supplement: Supplementary file 4 — Supplementary Figure S3a–c. [file 41598_2021_86520_MOESM4_ESM.tif]

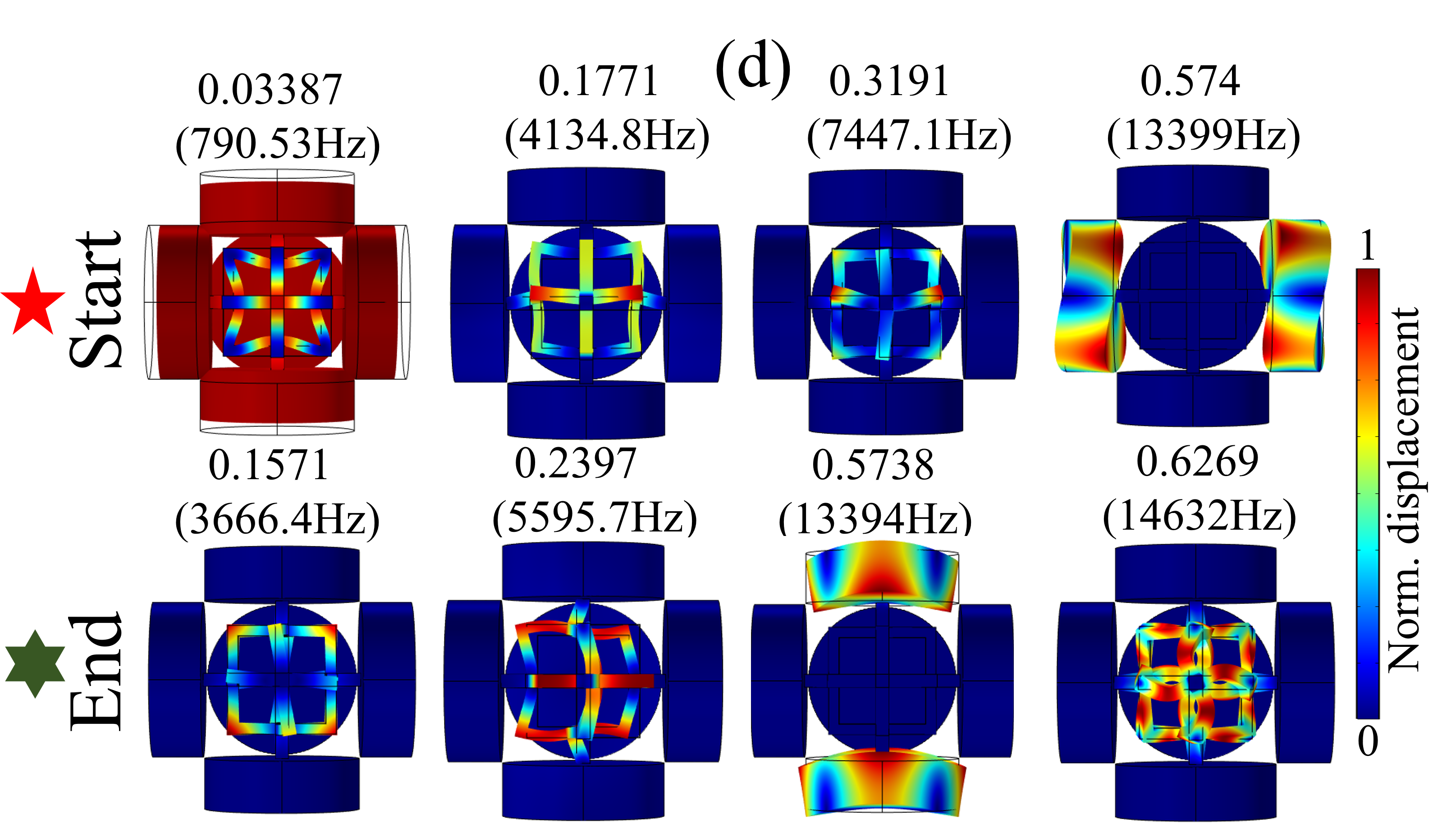

Supplement: Supplementary file 5 — Supplementary Figure S3d. [file 41598_2021_86520_MOESM5_ESM.tif]

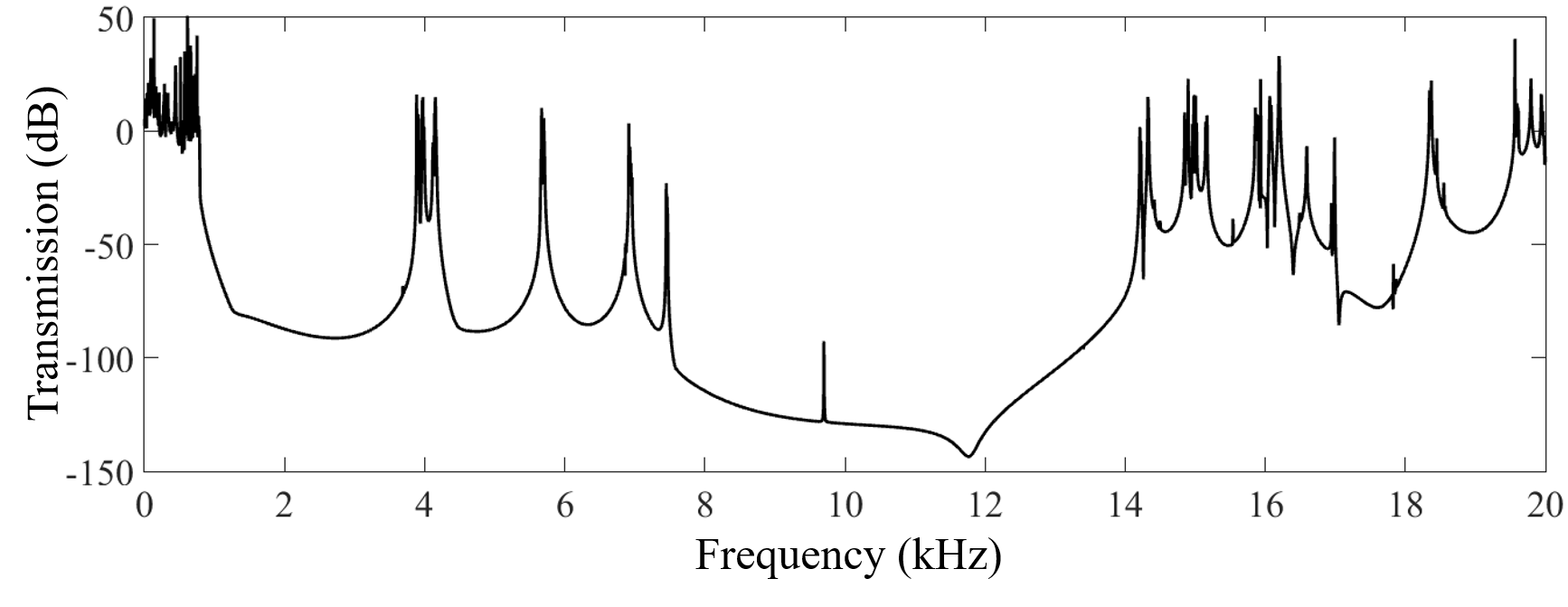

Supplement: Supplementary file 6 — Supplementary Figure S4. [file 41598_2021_86520_MOESM6_ESM.tif]

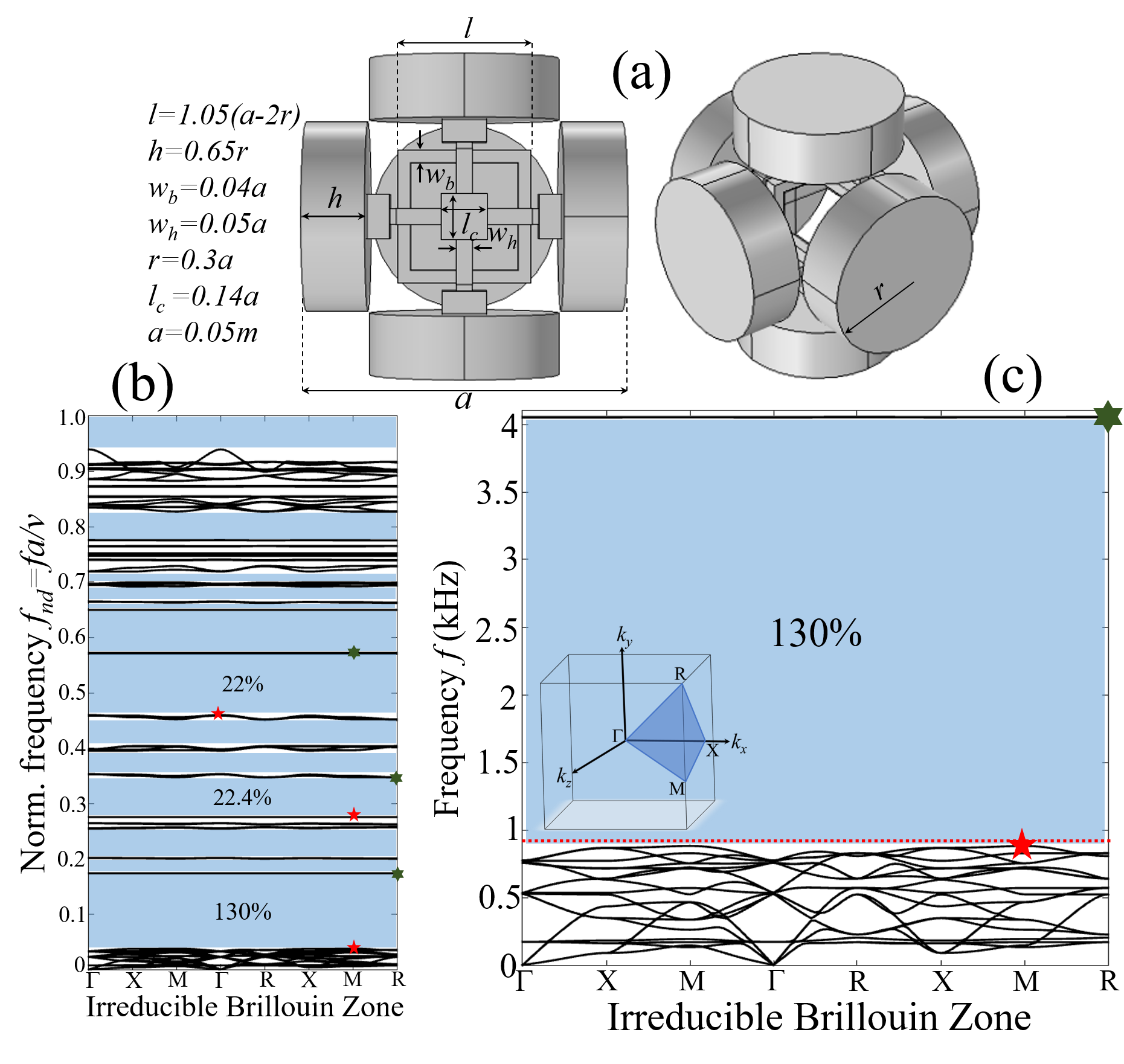

Supplement: Supplementary file 7 — Supplementary Figure S5a–c. [file 41598_2021_86520_MOESM7_ESM.tif]

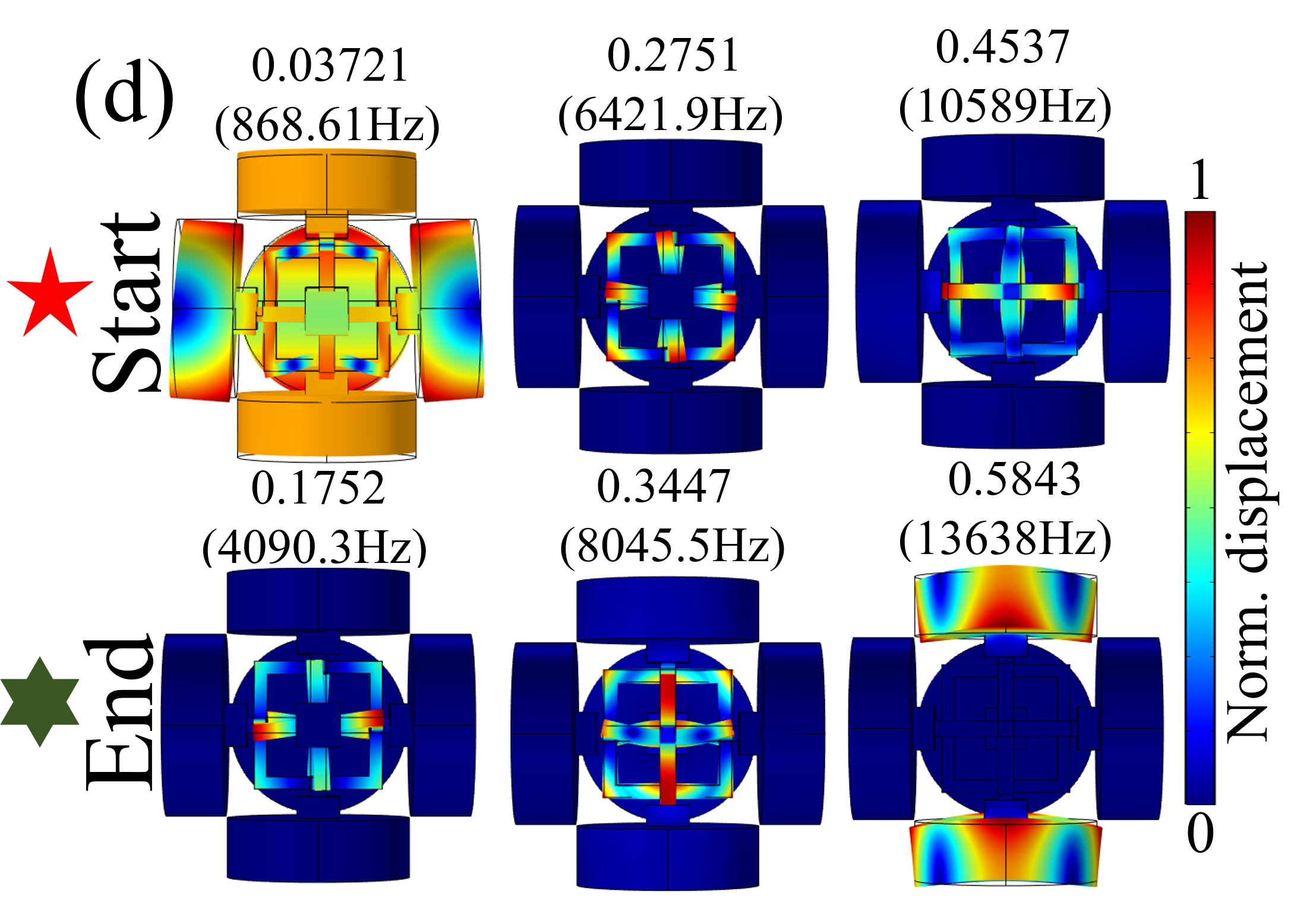

Supplement: Supplementary file 8 — Supplementary Figure S5d. [file 41598_2021_86520_MOESM8_ESM.tif]

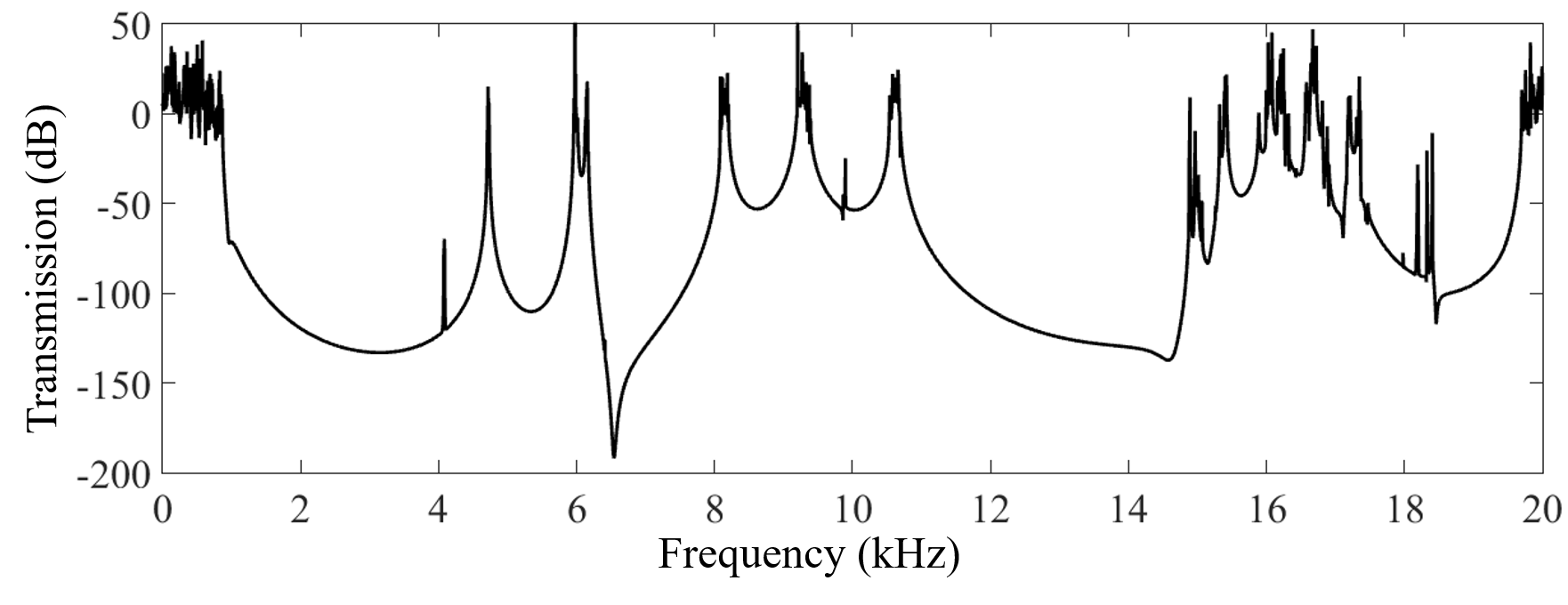

Supplement: Supplementary file 9 — Supplementary Figure S6. [file 41598_2021_86520_MOESM9_ESM.tif]

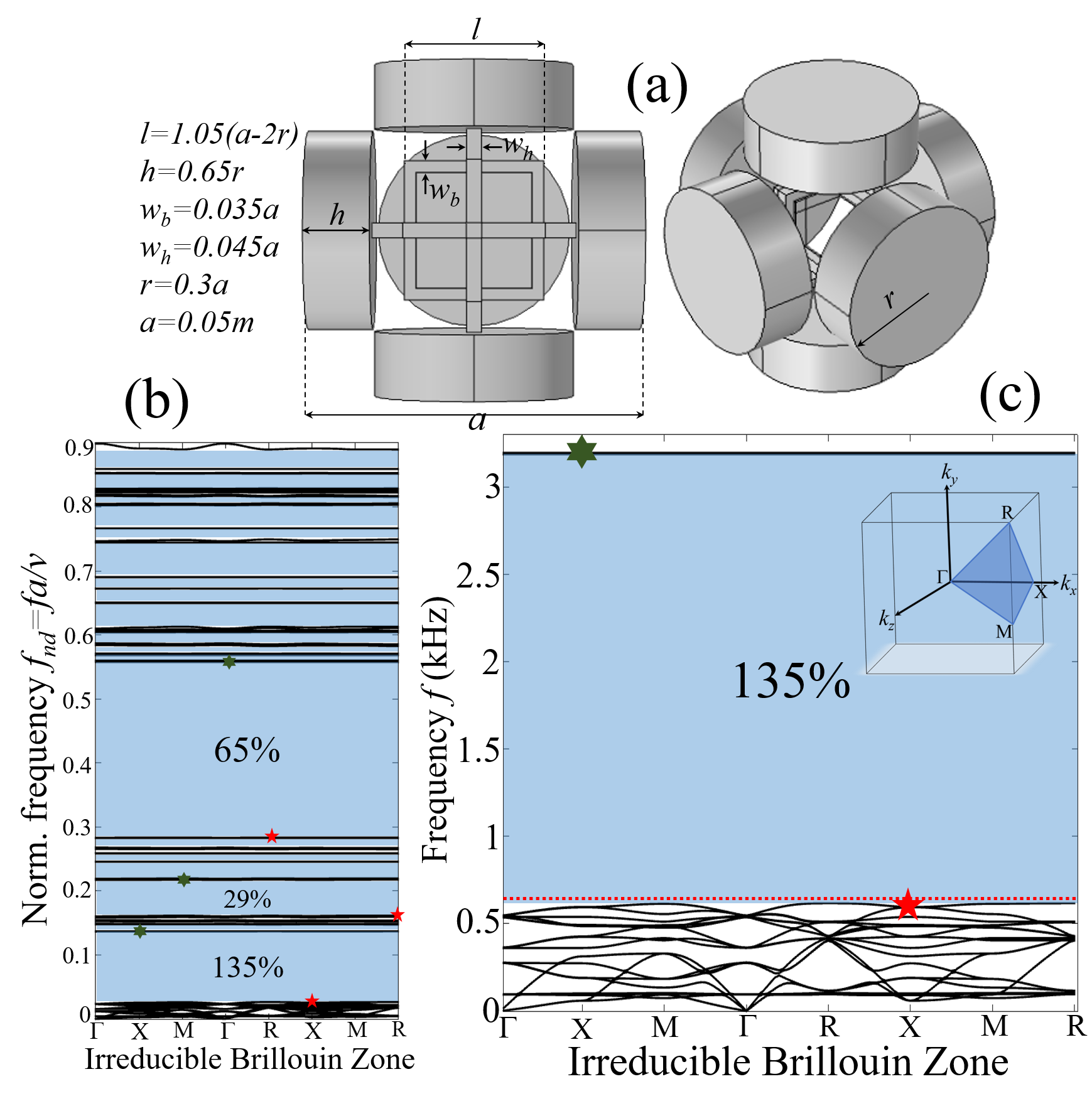

Supplement: Supplementary file 10 — Supplementary Figure S7a–c. [file 41598_2021_86520_MOESM10_ESM.tif]

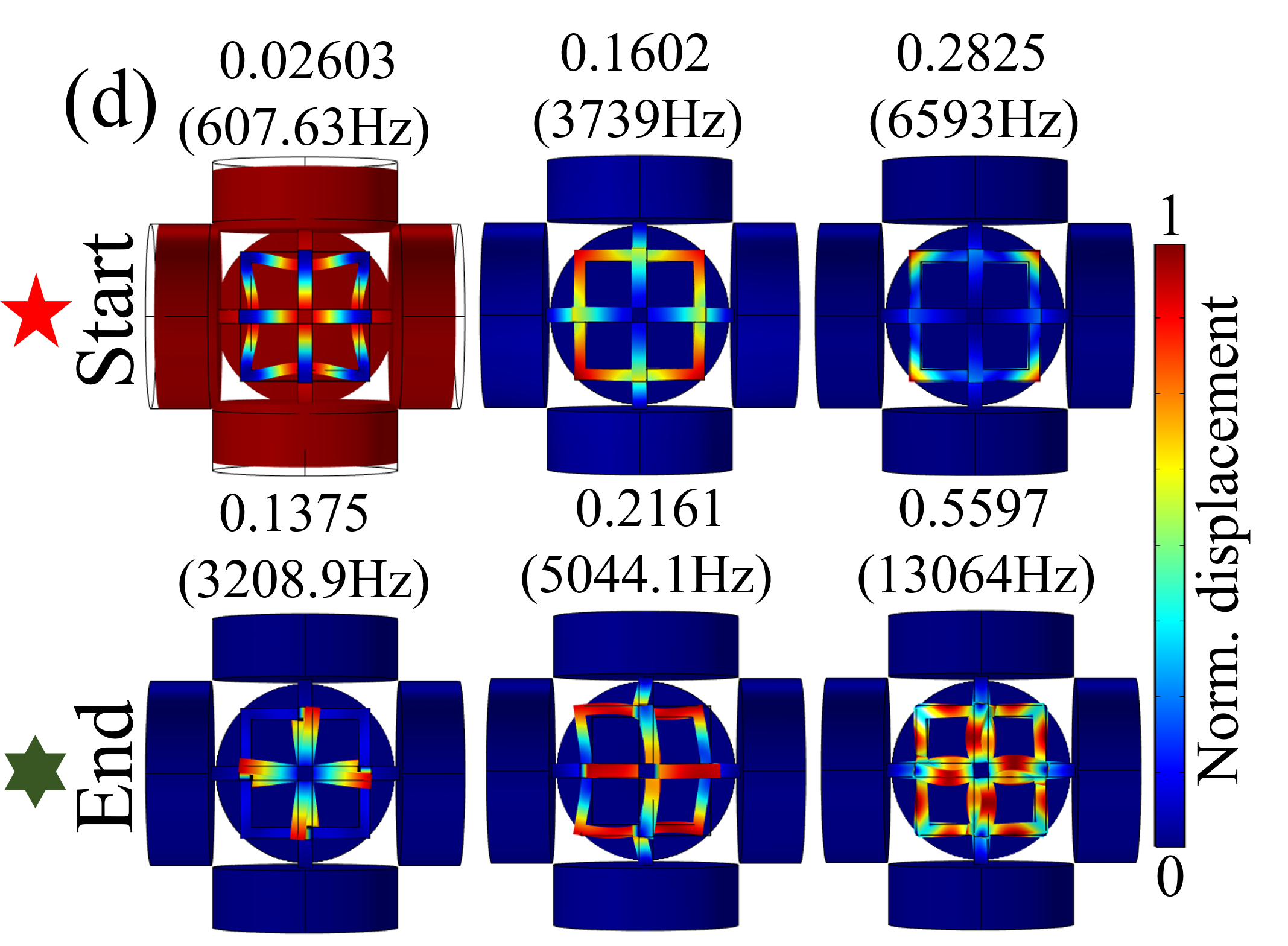

Supplement: Supplementary file 11 — Supplementary Figure S7d. [file 41598_2021_86520_MOESM11_ESM.tif]

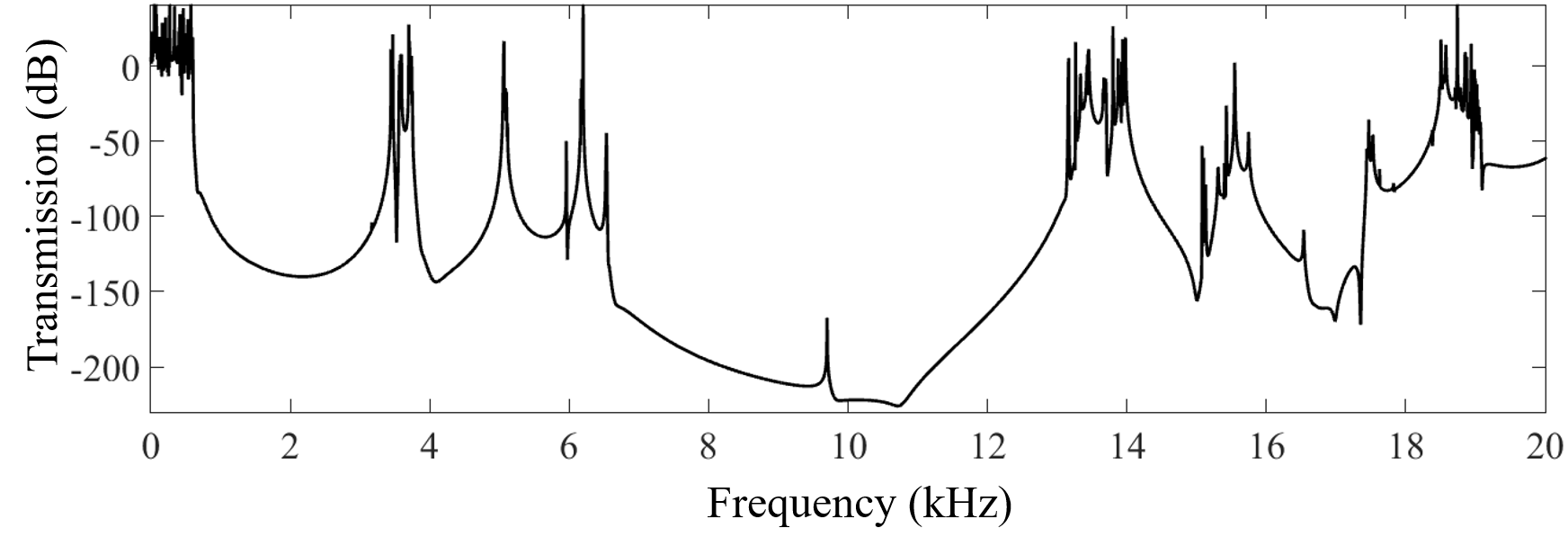

Supplement: Supplementary file 12 — Supplementary Figure S8. [file 41598_2021_86520_MOESM12_ESM.tif]

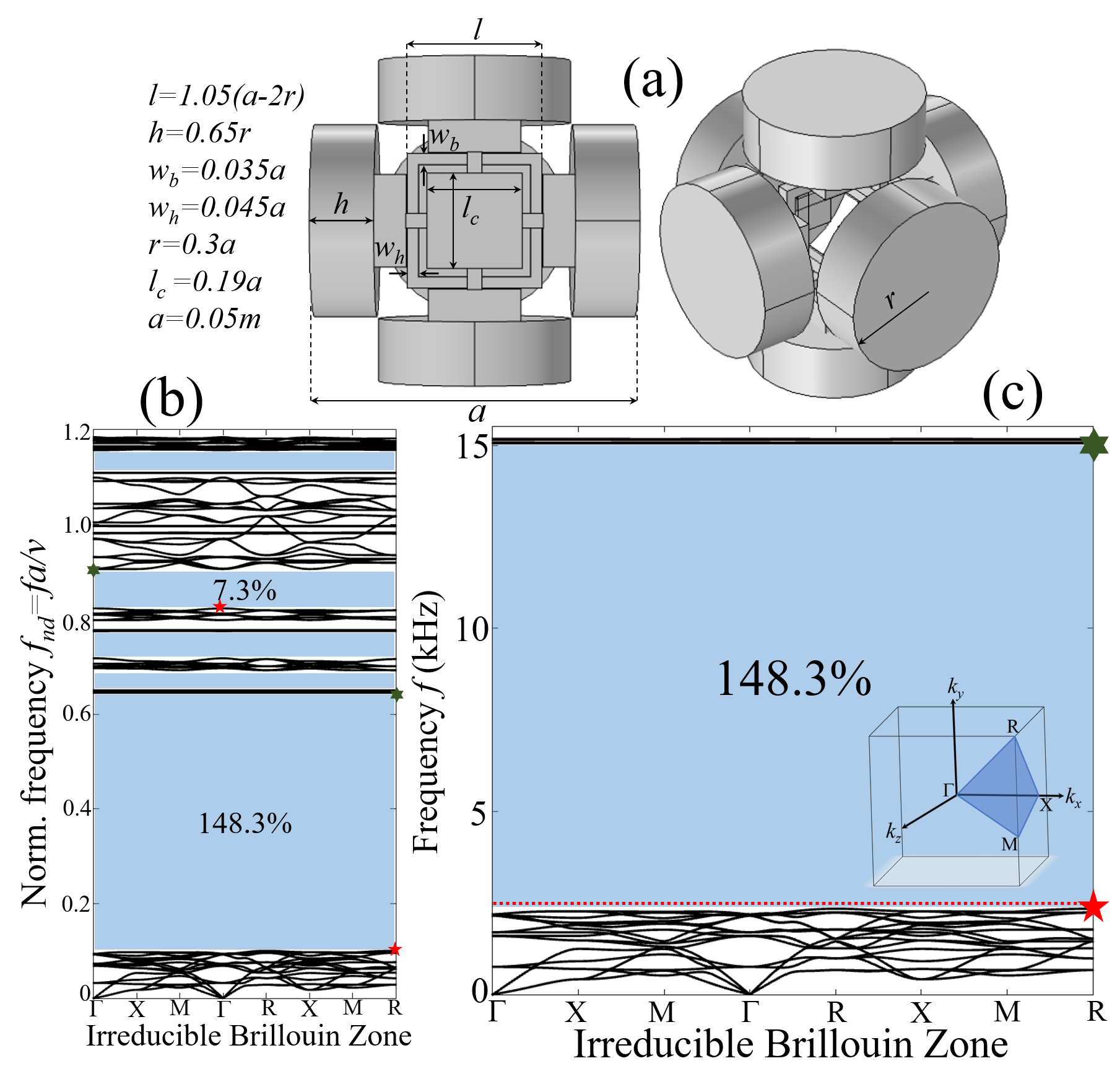

Supplement: Supplementary file 13 — Supplementary Figure S9a–c. [file 41598_2021_86520_MOESM13_ESM.tif]

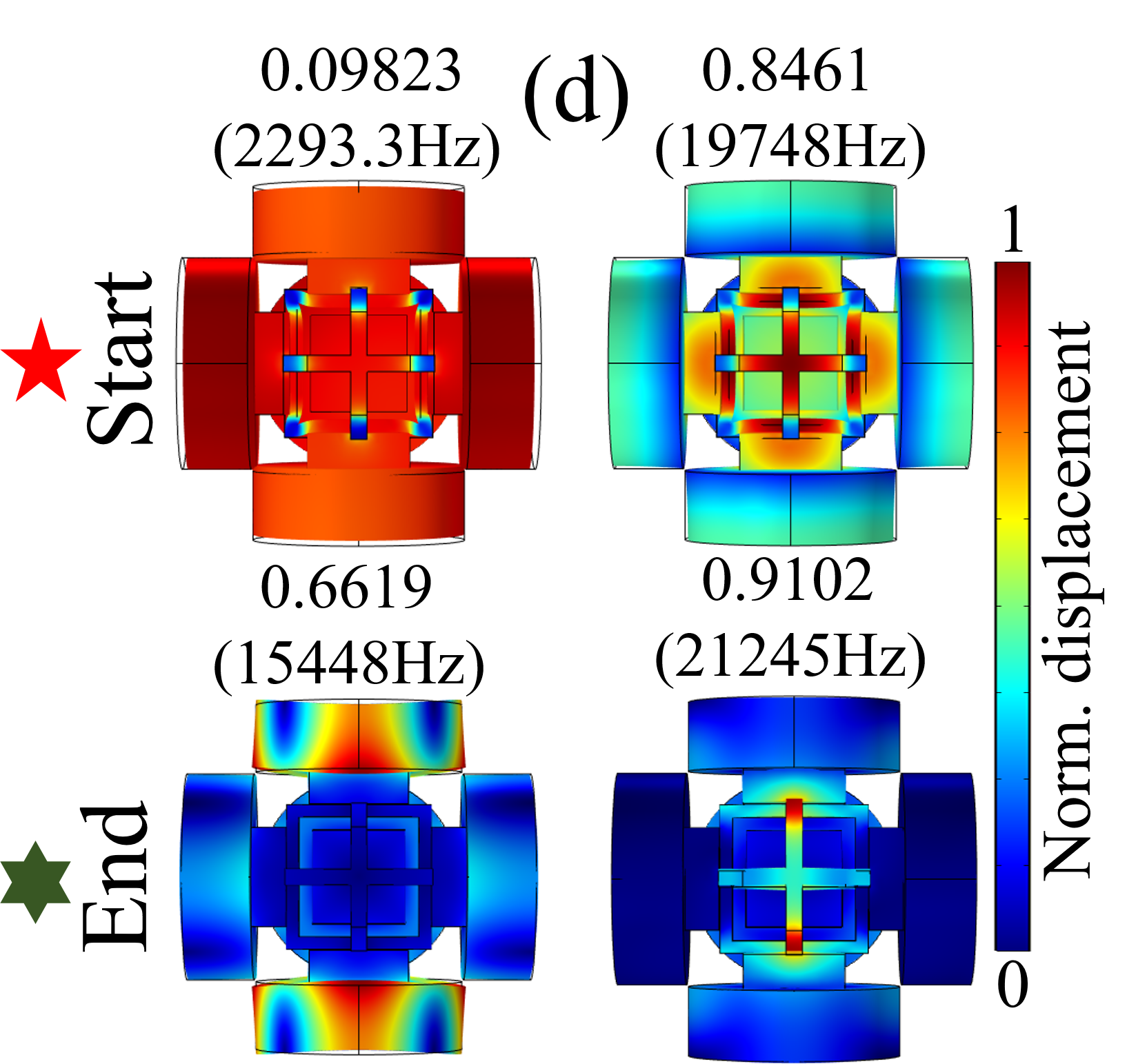

Supplement: Supplementary file 14 — Supplementary Figure S9d. [file 41598_2021_86520_MOESM14_ESM.tif]

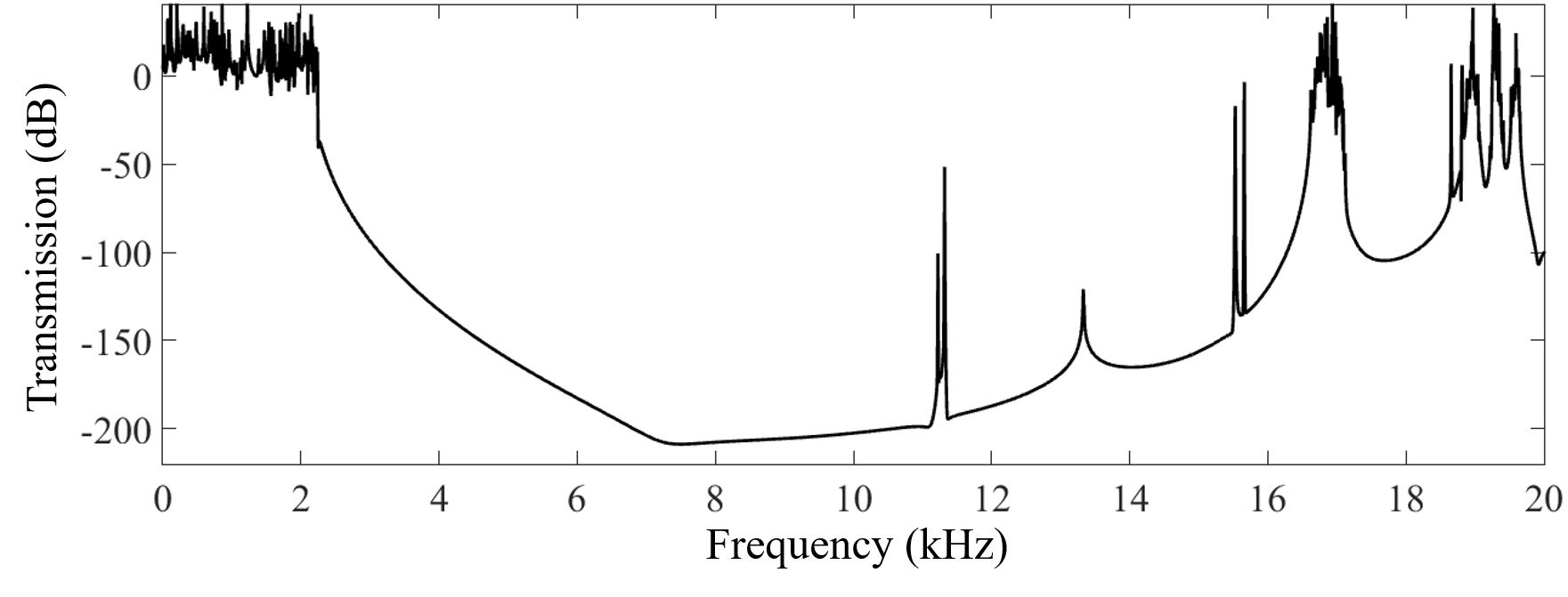

Supplement: Supplementary file 15 — Supplementary Figure S10. [file 41598_2021_86520_MOESM15_ESM.tif]

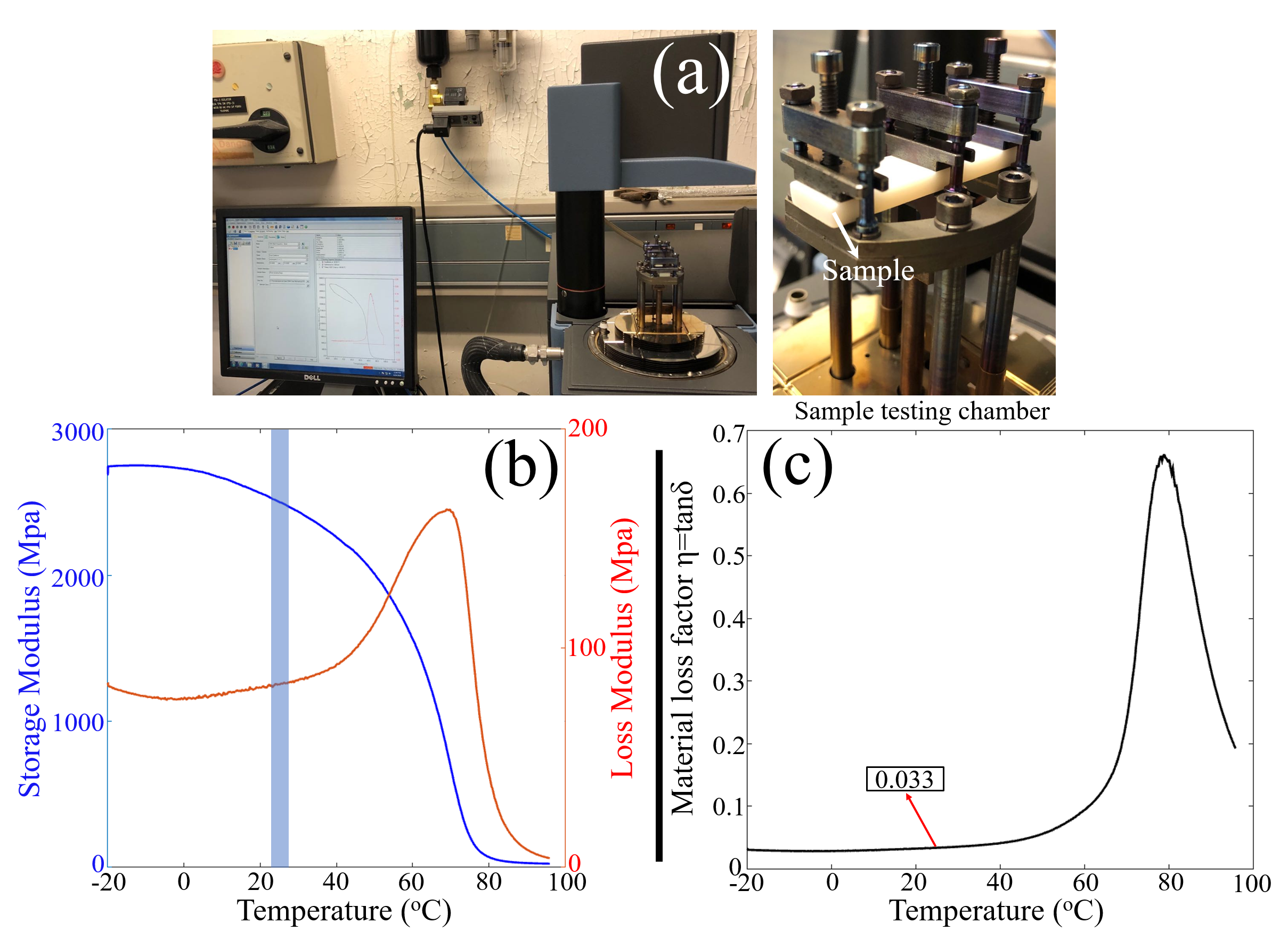

Supplement: Supplementary file 16 — Supplementary Figure S11. [file 41598_2021_86520_MOESM16_ESM.tif]
